# A Novel Anticancer Agent, 8-Methoxypyrimido[4′,5′:4,5]thieno(2,3-*b*) Quinoline-4(3H)-One Induces Neuro 2a Neuroblastoma Cell Death through p53-Dependent, Caspase-Dependent and -Independent Apoptotic Pathways

**DOI:** 10.1371/journal.pone.0066430

**Published:** 2013-06-18

**Authors:** Upasana Sahu, Himakshi Sidhar, Pankaj S. Ghate, Gopal M. Advirao, Sathees C. Raghavan, Ranjit K. Giri

**Affiliations:** 1 Division of Molecular and Cellular Neuroscience, National Brain Research Centre, Manesar, Haryana, India; 2 Department of Biochemistry, Kuvempu University, Davanagere, Karnataka, India; 3 Department of Biochemistry, Indian Institute of Science, Bangalore, Karnataka, India; University of Colorado, United States of America

## Abstract

Neuroblastoma is the most common cancer in infants and fourth most common cancer in children. Despite recent advances in cancer treatments, the prognosis of stage-IV neuroblastoma patients continues to be dismal which warrant new pharmacotherapy. A novel tetracyclic condensed quinoline compound, 8-methoxypyrimido [4′,5′:4,5]thieno(2,3-*b*) quinoline-4(3H)-one (MPTQ) is a structural analogue of an anticancer drug ellipticine and has been reported to posses anticancer property. Study on MPTQ on neuroblastoma cells is very limited and mechanisms related to its cytotoxicity on neuroblastoma cells are completely unknown. Here, we evaluated the anticancer property of MPTQ on mouse neuro 2a and human SH-SY5Y neuroblastoma cells and investigated the mechanisms underlying MPTQ-mediated neuro 2a cell death. MPTQ-mediated neuro 2a and SH-SY5Y cell deaths were found to be dose and time dependent. Moreover, MPTQ induced cell death reached approximately 99.8% and 90% in neuro 2a and SH-SY5Y cells respectively. Nuclear oligonucleosomal DNA fragmentation and Terminal dUTP Nick End Labelling assays indicated MPTQ-mediated neuro 2a cell death involved apoptosis. MPTQ-mediated apoptosis is associated with increased phosphorylation of p53 at Ser15 and Ser20 which correlates with the hyperphosphorylation of Ataxia-Telangiectasia mutated protein (ATM). Immunocytochemical analysis demonstrated the increased level of Bax protein in MPTQ treated neuro 2a cells. MPTQ-mediated apoptosis is also associated with increased activation of caspase-9, -3 and -7 but not caspase-2 and -8. Furthermore, increased level of caspase-3 and cleaved Poly (ADP Ribose) polymerase were observed in the nucleus of MPTQ treated neuro 2a cells, suggesting the involvement of caspase-dependent intrinsic but not extrinsic apoptotic pathway. Increased nuclear translocation of apoptosis inducing factor suggests additional involvement of caspase-independent apoptosis pathway in MPTQ treated neuro 2a cells. Collectively, MPTQ-induced neuro 2a cell death is mediated by ATM and p53 activation, and Bax-mediated activation of caspase-dependent and caspase-independent mitochondrial apoptosis pathways.

## Introduction

Neuroblastoma is an aggressive, heterogeneous and most common extra cranial childhood tumour affecting approximately 1 in 7000 children [1], [2]. It originates from neuronal precursor cells of sympathetic lineage and gives rise to tumours along sympathetic nervous system [3], [4]. Most primary tumours occur within the abdomen with at least 50% arising in the adrenal medulla. Other common sites of tumour development are the neck, chest, abdomen and pelvis [5], [6]. Although rare, brain also appears to be the site for primary neuroblastomas in approximately 5% of European Neuroblastoma study group [7], [8]. Neuroblastomas are classified into four different stages (I–IV). Complete regression of the disease with minimal therapy is seen in most infants with stages I or II even with metastatic disease but older patients with stages III or IV frequently have metastatic disease that grows relentlessly, despite the use of intensive multimodal therapy [5]. Furthermore, older neuroblastoma patients with stage IV are at high risk for death from refractory disease [3]. A similar observation was also reported in India. Only 2 out of 101 neuroblastoma patients with stage III or IV remain disease free and in others, disease relapsed soon after completing the therapy (patients were administered courses of “OPEC” therapy, namely, vincristine 1.5 mg/m^2^ and cyclophosphamide 600 mg/m^2^ on day 1, cisplatin 60 mg/m^2^ on day 2 and etoposide 120 mg/m^2^ on day 4) indicating the negligible effect of these chemotherapeutic agents [9]. Therefore, identification and study on the development of new drugs to treat high-risk neuroblastoma are warranted.

Cancer cells are mitotic. Multiple cellular pathways play pivotal roles in the proliferation of cancer cells through continuous DNA replication, a key event in cancer cell proliferation. Therefore, many known therapeutic drugs were developed against the machineries that replicate DNA. DNA interacts with various DNA intercalating drugs and inhibits cell proliferation. One such drug is ellipticine. Ellipticine (5,11-dimethyl-6H-pyrido[4,3-b] carbazole) is an alkaloid, first isolated from the leaves of Ochrosis elliptica in 1959 [10]. Ellipticine has been successfully used to treat various types of cancer such as acute myeloblastic leukemia, osteolytic breast cancer metastasis, kidney cancers, brain tumours [11] and neuroblastoma [12], [13], [14] with limited toxic side effects and complete lack of haematological toxicity [15]. However, ellipticine resistant neuroblastoma cells have also been reported [16]. Since, high-risk neuroblastomas develop resistance to cytostatics; development of new drugs to handle these solid tumours is a constant need. Many derivatives of ellipticine and structural analogue of ellipticine were developed and tested for DNA intercalating property similar to ellipticine. One such compound, 8-methoxypyrimido[4′,5′:4,5]-thieno(2,3-b)quinoline-4(3H)-one (MPTQ), containing tetracyclic condensed quinoline ring system was developed [17] and was shown to intercalate with calf thymus DNA [18]. MPTQ has been shown to exhibit its cytotoxic effect on human promyelocytic leukemia HL-60, melanoma B16F10 cells [19] and induces apoptosis in a wide range of leukemic cell lines [20], [21]. Mechanisms related to MPTQ-mediated cancer cell death are limited. Recently a single report has suggested the MPTQ-mediated leukemic cell death involved activation of p53-dependent intrinsic and extrinsic apoptosis pathway [21]. However, the literature on the effect of MPTQ on neuroblastoma is limited, only with a hint of cytotoxicity [19] and mechanisms related to MPTQ-mediated neuroblastoma cytotoxicity are completely unknown.

To study this in detail, we designed experiments to check the cytotoxic effect of MPTQ on neuro 2a and SH-SY5Y (a mouse and a human neuroblastoma cell line respectively). MPTQ–mediated neuro 2a and SH-SY5Y cell death is dose and time-dependent. Almost all neuro 2a cells (∼99.8%) were found dead after 96 hours (hrs) upon 30 µM of MPTQ treatment and approximately 90% of SH-SY5Y cells died within 6 days of 90 µM of MPTQ treatment. Since neuro 2a cells showed faster response to MPTQ treatment than SH-SY5Y cells, we utilized neuro 2a cells to study molecular mechanisms involved in MPTQ-mediated neuroblastoma cell death. Nuclear DNA fragmentation and Terminal dUTP Nick End Labelling (TUNEL) assays demonstrated significant induction of DNA double-strand breaks (DSBs) in neuro 2a cells after 48 hours of MPTQ treatment indicating the involvement of apoptosis. Molecular analysis of MPTQ-mediated cell death in neuro 2a cells demonstrated the involvement of Ataxia-Telangiectasia mutated (ATM) activation (DNA double strand break marker), p53 activation (DNA damage marker), Bax upregulation (mitochondrial apoptosis marker), caspase and Poly(ADP Ribose) polymerase (PARP)-dependent activation of intrinsic but not extrinsic apoptotic pathway. Our results also demonstrated the involvement of apoptosis inducing factor (AIF) suggesting the activation of caspase-independent apoptotic pathway in MPTQ-mediated neuroblastoma cell death. Collectively, our results for the first time addressed multiple mechanisms associated with MPTQ-mediated neuroblastoma cell deaths and suggest the possible use of MPTQ in neuroblastoma therapy.

## Materials and Methods

### Cell Lines and Culture Condition

Neuro 2a (CCL-131), a mouse neuroblastoma cell line and SH-SY5Y, a human neuroblastoma cell line were obtained from Prof. Nihar Ranjan Jana [22], [23] and Dr. Pankaj Seth [24] NBRC, Manesar, Haryana, India respectively. Neuro 2a cells were grown in DMEM (Invitrogen, USA) containing 5% heat inactivated fetal bovine serum (FBS) (Hyclone, USA), 100 U/ml penicillin and 100 mg/ml streptomycin (Invitrogen, USA) in a humidified incubator at 37°C with 5% CO_2_ in air. SH-SY5Y cells were grown in DMEM containing 20% heat inactivated FBS, 100 U/ml penicillin and 100 mg/ml streptomycin in a humidified incubator at 37°C with 5% CO_2_ in air.

### Preparation of MPTQ Reagent

MPTQ synthesis and characterization has been reported earlier [17]. MPTQ stock solution of 90 mM was prepared in cell culture grade dimethylsulfoxide (Sigma-Aldrich, USA) and sonicated at 75% energy for 2 minutes (mins) with 15 seconds (secs) on and 10 secs off cycles. MPTQ of 7.5, 15, 30 and 60 mM were prepared from 90 mM stock by diluting further in DMSO. Working concentrations of MPTQ were prepared by 3000-fold dilution of the stock solutions in DMEM containing 5% FBS for neuro-2a cells and 1000-fold dilution of stock reagents in DMEM containing 20% FBS for SH-SY5Y cells.

### Antibodies

Rabbit anti-phospho-p53 (Ser15) antibody (9384), mouse anti-p53 antibody (1C12, monoclonal; 2524), rabbit anti-cleaved PARP antibody (9544), rabbit anti-caspase-3 antibody (9662), rabbit anti-caspase-6 antibody (9762), rabbit anti-caspase-7 antibody (9492) and mouse anti-caspase-9 antibody (C9, monoclonal; 9508) were purchased from Cell Signaling (Cell Signaling Technology Inc., USA). Mouse anti-GAPDH antibody (6C5, monoclonal; SC32233), mouse anti-PARP-1 antibody (C2-10, monoclonal; S53643), rabbit anti-phopho-p53 (Ser20) antibody (SC-21872-R), Goat anti-AIF antibody (SC-9416) and anti-Bax antibody (SC-526) were purchased from Santa Cruz Biotechnology (Santa Cruz, USA). Rabbit anti-AIF antibody (IMG-303-2) and rabbit anti-phospho-ATM (Ser1981) antibody (IMG-90221-1) were purchased from Imgenex (Imgenex Corp., USA). Rabbit anti-caspase-2 antibody (AF826) and rabbit anti-caspase-8 antibody (AF1650) were purchased from R&D systems (R&D Systems, Inc., USA). Horse-radish peroxidase (HRP)-conjugated secondary antibodies against mouse, goat and rabbit were purchased from Pierce (Thermo Scientific, USA) and were used for immunoblot analysis. Alexa fluor 594 conjugated goat anti-rabbit F(ab’)^2^ fragments and Alexa fluor 594 goat anti-mouse F(ab’)^2^ fragments were purchased from Molecular Probes (Invitrogen, USA) and were used in immunocytochemical analysis.

### Live-Dead Assay

MPTQ-mediated cell death in neuro 2a and SH-SY5Y was studied using Live-Dead assay kit (Invitrogen, USA). The kit has two fluorescent dyes, calcein-AM and ethidium homodimer. In principle, calcein-AM can enter any cells but labels only live cells. It is converted by cellular cytoplasmic esterases to a highly green fluorescent calcein. Ethidium homodimer is excluded by live cells with intact membrane but enters dead cell with broken membrane to stain their nuclei red. Therefore, live cells fluoresce green where as dead cells fluoresce red. Neuro 2a and SH-SY5Y cells were seeded at 20,000 cells/well and 100,000 cells/well respectively in 24-well tissue culture plates. After 48 hrs, neuro 2a cells were treated with 2.5, 5, 10, 20 and 30 µM of MPTQ whereas SH-SY5Y cells were treated with 90 µM of MPTQ in their respective cell culture medium. Control cells were treated with equal volume of DMSO. Live dead assay was performed 24 hrs post treatment in neuro 2a cells but after 4days in SH-SY5Y cells. Live-Dead reagent was diluted either in serum free DMEM or PBS (1X) to 2X concentration and added directly to the cell culture media at 1∶1 ratio followed by a gentle but thorough mixing. Cells were incubated in dark for 30 minutes at room temperature. After incubation, 3–4 random images were captured per well using an inverted microscope (Nikon Ti Eclipse, Japan) supported by quantis monochromatic cooled CCD camera, MetaMorph software and using FITC (for calcein) and Texas Red (for ethidium homodimer) filter cubes. Number of live (green) and dead (red) cells were counted using the multi-wavelength cell scoring module of MetaMorph software. For time-dependent cytotoxicity of MPTQ on neuroblastoma cells, neuro 2a cells were treated with 30 µM of MPTQ for 24, 48, 72 and 96 hrs and Live-Dead assay was performed as described above.

### Cell Viability Test by MTT Utilization Assay

Cell viability was analyzed by 3-(4,5-dimethylthiazol-2-yl)-2,5-diphenyl-terazolium bromide (MTT) dye (Sigma-Aldrich, USA) utilization and its conversion to formazan, as described previously [25] with little modification. Briefly, 20,000 neuro 2a cells or 100, 000 SH-SY5Y cells were seeded per well in a 24-well plate. After 48 hrs, neuro 2a cells were treated with 30 µM of MPTQ for 24, 48 and 72 hrs in triplicates whereas SH-SY5Y cells were treated with 30, 60 and 90 µM of MPTQ for 2, 4 and 6 days in triplicates. DMSO treated cells were used as controls. After treatment, media were replaced with serum free DMEM media containing 1 mg/ml of MTT and incubated at 37°C in a 5% CO_2_ incubator for additional 4 hrs. Intracellular formazan products were solubilised by replacing MTT reagent with MTT solvent (4 mM HCl and 0.1% NP40 in isopropanol) and incubated for 15 minutes at 37°C in 5% CO_2_. Contents were transferred to a 96-well tissue culture plate and optical density was measured at 570 nm with reference wavelength at 620 nm using Magellan-6 (version 6.5) software provided with the plate reader (TECAN sunrise, Austria). Only MTT solvent was used as reference. The cytotoxicity caused by MPTQ was expressed as amount of formazan produced and compared to DMSO treated controls.

### DNA Fragmentation Assay

Neuro 2a cells were seeded at a density 20,000 cells/well in a 24-well tissue culture plate. After 48 hrs of culture, cells were incubated with 2.5, 5, 10, 20 or 30 µM of MPTQ in triplicates. DMSO treated cells were used as controls. Cells were lysed after 48 hrs of treatment in 100 µl of digestion buffer (100 mM NaCl; 10 mM Tris Cl, pH 8.0; 1 mM EDTA, pH 8.0; 0.1% SDS and 0.1 mg/ml Proteinase-K) per sample, incubated at 50°C for 3 hrs followed by addition of 5 µl of SDS-OUT reagent from Pierce (Thermo technology, USA). The content was mixed and incubated on ice for 20 minutes followed by centrifugation at 10,000×g for 10 minutes at 4°C. Supernatants were collected in fresh centrifuge tubes, treated with RNase for 20 minutes on ice followed by quantification using NanoVue plus spectrophotometer (GE healthcare, UK). Equal amount of DNA from each sample (1.5 µg) were electrophoresed in a 2% agarose gel. Images of DNA agarose gels were captured using chemidoc XRS^+^ geldoc system (BIORAD, USA). Densitometric analysis of the fragmented DNA was done using ImageLab software by subtracting the high molecular weight DNA density from total DNA density.

### Apoptosis Analysis by TUNEL Assay

Dead End Fluoremetric TUNEL system kit (Promega corp, USA) was used to detect nuclear DNA fragmentation in MPTQ treated neuroblastoma cells, a molecular measure of apoptosis. The kit detects DNA strand breaks at single cell level by incorporating fluorescein-12-dUTP at free 3′-OH DNA ends by recombinant terminal deoxynucleotidyl transferase (rTdT). Neuroblastoma cells were seeded in a density of 5×10^5^ cells/25 cm^2^ tissue culture flasks and cultured for two days followed by treatment with 30 µM of MPTQ. Cells treated with equal amount of DMSO were used as controls. The assay was done in suspension. After 48 hrs of treatment, cells were collected in 1.5 ml micro-centrifuge tubes by gentle scrapping followed by centrifugation at 300×g for 5 mins at room temperature (RT). Cell pellets were washed two times with sterile PBS by centrifugation at 300×g for 5 mins at RT. Cells pellets were dislodged and fixed with 4% Paraformaldehyde for 30 mins at RT followed by two PBS (phosphate buffer saline) washes for 5 mins at RT. Cells were permeabilized with 0.2% Triton X-100 at room temperature for 5 mins and washed twice with PBS for 5 mins at RT. Cells were then labelled with fluorescein-12-dUTP as described by manufacturer’s protocol. At this stage, a positive and negative control samples were prepared from untreated neuro 2a cells. Untreated but fixed cells were treated with DNaseI (amplification grade; Invitrogen, USA) to a final concentration of 7 U/ml for 15 mins at RT followed by three PBS washes at RT for 5 min each. Cells were then aliquoted into two. Both the aliquots were then labelled with fluorescein-12-dUTP either in the presence of rTdT (positive control) or in the absence of rTdT (negative control). Labelled and washed cells were then spotted on slides coated with tissue adhering solution, allowed to dry and then mounted with anti-fade gold mounting media containing DAPI (Invitrogen, USA). After 16 hrs of curing, fluorescein-12-dUTP-labelled DNA was visualized and images were captured from several random fields as mentioned before. Images were analyzed for percentage of TUNEL positive cells using cell scoring module of MetaMorph software.

### Bright Field Imaging

Neuro 2a and SH-SY5Y cells were grown in 24 well plates and either treated with 30 µM of MPTQ or with equivalent amount of DMSO (control). Bright field images of neuro 2a and SH-SY5Y cells were captured after 48 hrs and 6 days of treatment respectively from at least 3 random fields using 40X objective lens with Hoffman modulation and thickness correction rings in Nikon TS100 inverted microscope using NIS elements BR (2.3 version) software. Image scales were burned onto each picture using the same software.

### Immunocytochemistry

To determine key molecular players involved in MPTQ-mediated apoptosis in neuro 2a cells, we examined the nuclear level of phosphorylated p53 at Ser15 and Ser20, phosphorylated ATM at Ser1983, caspase-3, AIF and cleaved product of PARP and cytoplasmic level of pro-apoptotic Bcl2 family Bax protein in MPTQ treated and untreated neuro 2a cells by immunocytochemistry as described earlier [26]. Briefly, 20000 neuro 2a cells were seeded onto Poly-L-Lysine (0.05 mg/ml) coated cover glass slips or into each well of a 24 well cover glass plate. After 48 hrs, cells were treated with 30 µM of MPTQ or with equal amount of DMSO (controls) for 24 hrs. Cells were then washed with PBS and fixed with 4% PFA for 15 min. Next, the cells were permeabilized and nonspecific epitopes were blocked in blocking buffer (PBS with 0.1% BSA and 10% normal goat serum) for 1 hour at RT. Cells were then incubated with primary antibody in blocking buffer at 4°C overnight. After 5 washes in wash buffer (PBS with 0.1% BSA) for 5 mins each at RT, cells were incubated with appropriate secondary antibody conjugated to Alexa fluor 594 for 1 hour at RT. Cells were then washed five times in wash buffer (0.1% BSA in PBS), followed by one PBS wash and fixation with 4% PFA for 15 mins at RT. Cells were again washed thrice in PBS, rinsed once with water and mounted with anti-fade mounting medium containing DAPI. Fluorescent images were captured using 40X plan fluor or 60X plan apo (with oil) objective lens as described earlier using multi-dimension acquisition module of MetaMorph software. Images from a same batch were captured using identical image acquisition settings and preferably in one sitting. Z-stack images were deconvoluted using 3D-deconvolution module of MetaMorph. For qualitative presentation, maximum projections were generated from 12 in-focus planes and images were displayed with equal pixel intensity. For quantification of fluorescence intensity in nuclear or cytoplasmic compartments of cells, multi-wavelength cell scoring module was used with identical settings.

### Western Blotting

Neuro 2a cells were seeded in a density of 5×10^5^ cells/25 cm^2^ tissue culture flask or 1×10^6^ cells/75 cm^2^ tissue culture flasks and cultured for 2 days followed by treatment with 30 µM of MPTQ for 24 hrs. For experimental controls, cells were treated with equal volume of DMSO. After 24 hrs of treatment, cells were collected using cell scrapper and cell pellets were obtained by centrifugation at 1000 RPM followed by a PBS wash. Total cell lysates were obtained by dissolving cell pellets in 200 µl of RIPA buffer (150 mM Sodium chloride, 10 mM Tris-Cl (pH 7.5), 0.5% Triton X-100, 0.5% Sodium deoxycholate, 0.1% SDS, 1 mM Sodium Orthovanadate, 1 µl/ml of Protease inhibitor cocktail from Sigma-Aldrich). Lysates were sonicated at 75% energy for 2 mins with 15 secs on and 10 secs off cycle, cleared by centrifugation at 1000×g for 10 minutes at 4°C. Cytoplasmic and nuclear lysates were isolated as described earlier [27]. Protein concentration was determined by the bicinchoninic acid assay using Micro BCA Protein Estimation Kit (Thermo-Pierce, USA). Equal amount of proteins from each sample were size fractionated in appropriate SDS-PAGE gel and transferred onto 0.2 µm nitrocellulose membranes (BIORAD, USA). Blots were probed with anti-caspase-2, anti-caspase-8, anti-caspase-9, anti-caspase-3, anti-caspase-7, anti-PARP, anti-phospho-ATM and anti-AIF antibodies for overnight at 4°C. Activation of p53 was assessed using anti-p53, anti-phospho-p53 (Ser15) antibodies. Horseradish peroxidase-conjugated secondary antibodies were used to develop the membrane and visualisation of bands was performed using supersignal chemiluminiscent substrate from Pierce (Thermo-Pierce, USA). Anti-GAPDH either alone or in combination with anti-histone-H3 antibody was utilized to normalize protein loading and transfer.

### Statistical Analysis

Statistics were performed using Sigmastat 3.5 software. When 3 or more groups were compared simultaneously, one way analysis of variance (ANOVA) was used if data qualifies normality test. When data failed the normality test, nonparametric Kruskal-Wallis one way ANOVA on ranks was used. To compare two groups, Student’s t-test was employed using Microsoft EXCEL (two tailed, unpaired). Differences between groups were considered statistically significant when p≤0.05.

## Results

### MPTQ Induces Cytotoxicity in Neuro 2a Neuroblastoma Cells in a Dose-dependent Manner

**Figure 1:**
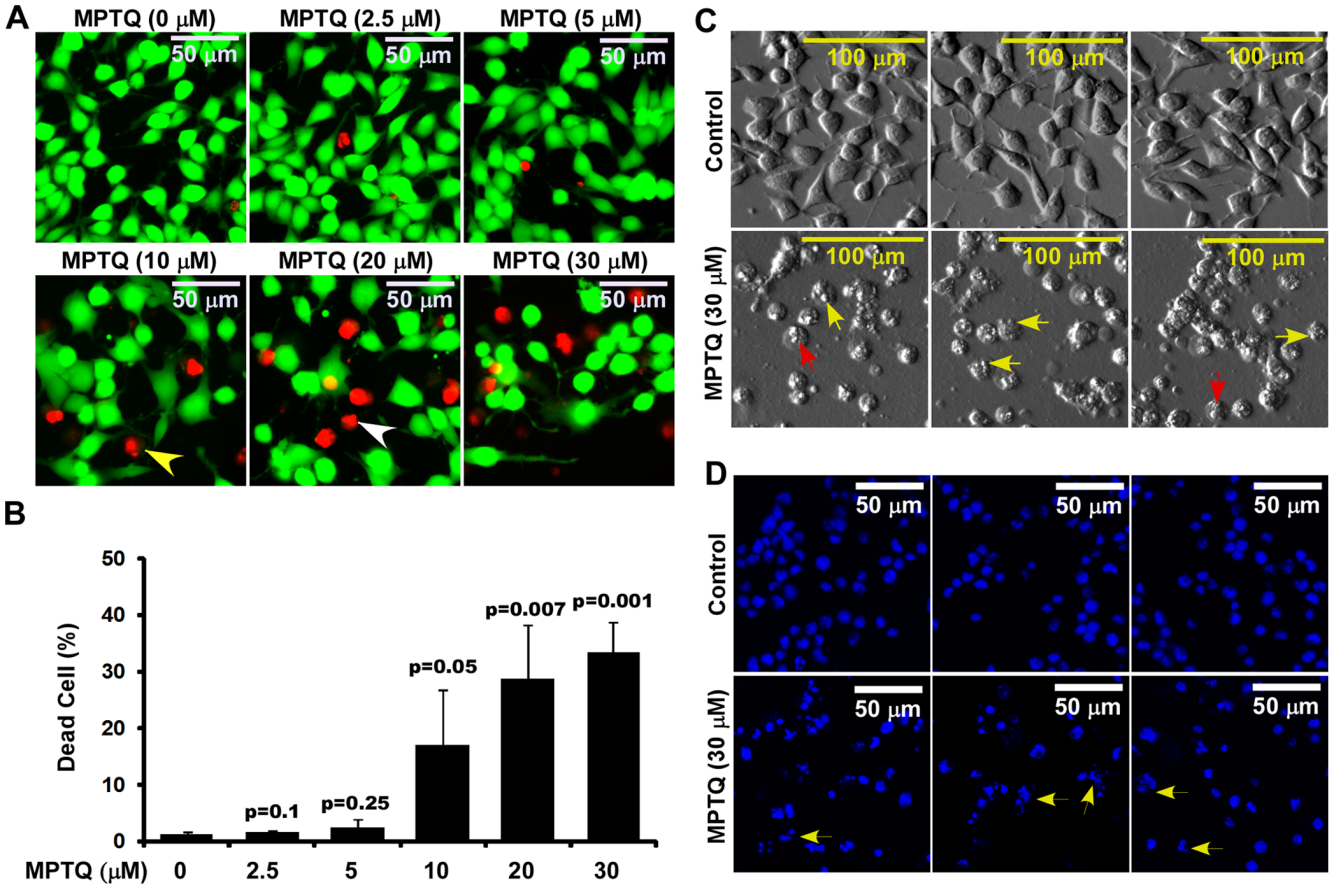
Increased cytotoxicity, and altered cell and nuclear morphology in MPTQ treated neuro 2a neuroblastoma cells. A) Live-Dead assay on neuro 2a neuroblastoma cells was performed after 24 hours of 2.5, 5, 10, 20 or 30 µM of MPTQ treatment. Cells treated with equal amount of DMSO and for same duration served as controls. Fluorescent images from four random fields were captured and were displayed with equal pixel intensity. B) Percentage of dead cells was calculated using multi-cell scoring module of MetaMorph software. Data are expressed as mean±standard deviation of three independent experiments. p value displayed for each treatment was calculated by comparing with control using Student’s t-test. p value ≤0.05 is considered significant. C) Comparative morphology of normal and MPTQ treated neuro 2a cells. Bright field images of normal and 30 µM of MPTQ treated neuro 2a cells demonstrate plasma membrane blebbing (yellow arrow), irregular nuclear compartments (red arrow) only in MPTQ treated cells but not in normal cells after 48 hrs of treatment. D) DAPI stained images exhibit condensed and fragmented nuclei (yellow arrow) only in MPTQ treated but not in normal cells, features typical to apoptotic cells.

Cytotoxic effect of MPTQ on neuro-2a neuroblastoma cells was studied at different doses of MPTQ utilizing Live-Dead assay. Neuro 2a cells were treated with 2.5, 5, 10, 20 or 30 µM of MPTQ for 24 hrs. Images obtained from three independent experiments clearly demonstrate dose-dependent increased cytotoxicity in MPTQ treated neuro 2a cells (Figure 1A). Images also demonstrate chromatin condensation (white arrow head) and nuclear fragmentation (yellow arrow head) in MPTQ treated and ethidium homodimer positive neuro 2a cells. Significant increase in cell deaths was observed from 10 to 30 µM of MPTQ treated neuro 2a cells than controls. Although the difference in cell death between 30 µM and 20 µM MPTQ treated cells was not significant but maximum cell deaths (33.2±5.5%) was observed in 30 µM of MPTQ treated cells (Figure 1B). Therefore, 30 µM of MPTQ treatment was used in our subsequent experiments on neuro 2a cells. Cell shrinkage, membrane blebbing, chromosomal condensation and DNA fragmentation are some of the characteristic morphological features of cells undergoing apoptosis [28], [29], [30]. To examine this, neuro 2a cell were treated with 30 µM of MPTQ for 48 hrs. Bright field images clearly demonstrated cell membrane blebbing (yellow arrow) and prominent compaction of nuclear compartment (red arrow) in MPTQ treated neuro 2a cell but not at all in control cells (Figure 1C). Furthermore, fluorescent images from DAPI (a nuclear stain) stained cells also demonstrated condensation as well as fragmentation of nuclei, as seen in apoptotic cells (Figure 1D). Thus, MPTQ-mediated neuro 2a cell death might involve apoptosis.

### Time-dependent Cytotoxic Effect of MPTQ on Neuro 2a Neuroblastoma Cells

**Figure 2:**
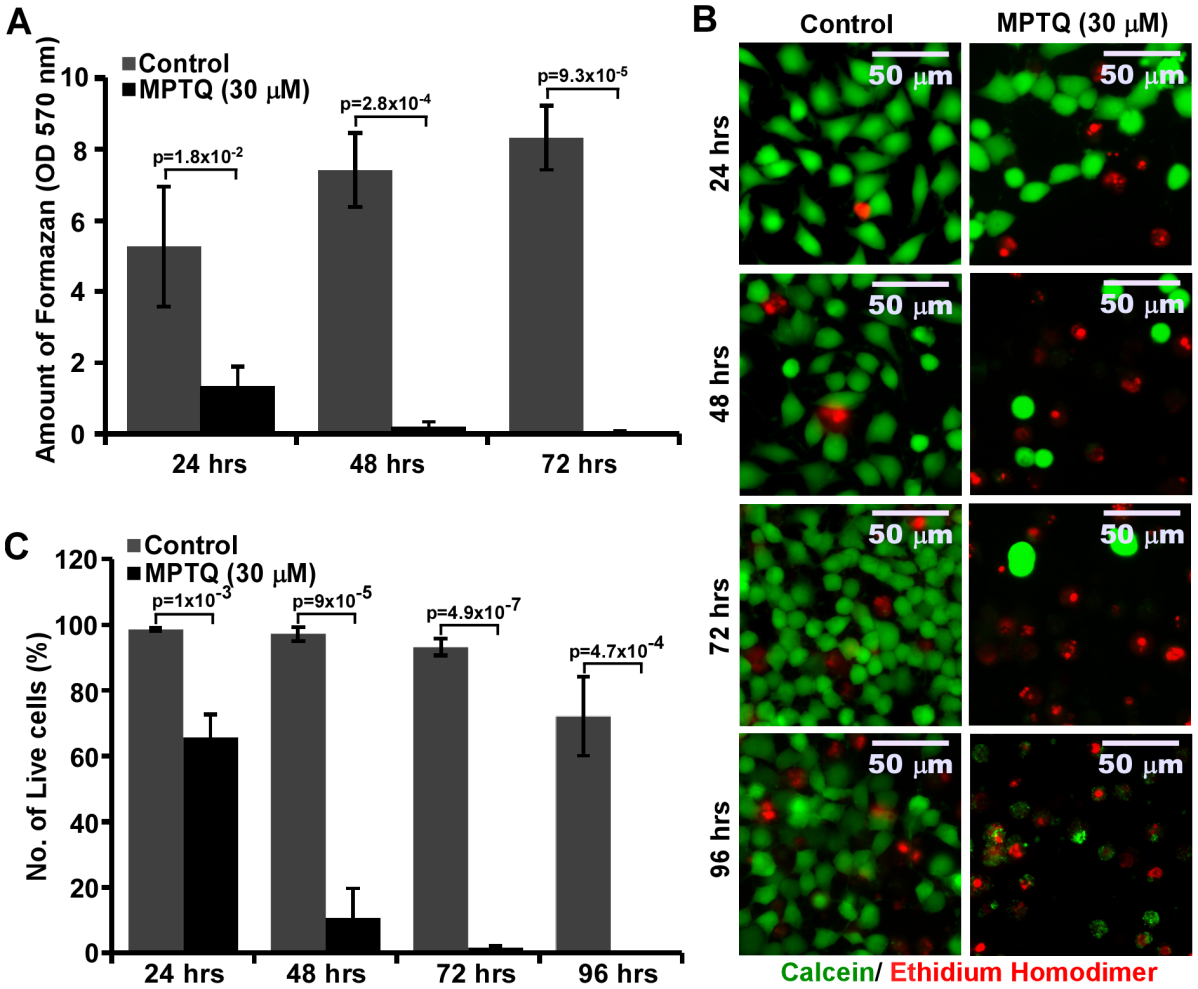
MPTQ treatment increases neuro 2a neuroblastoma cell death in a time-dependent manner using MTT and Live-Dead assays. Neuro 2a cells were treated with 30 µM of MPTQ. DMSO treated cells were considered as controls. A) After 24, 48 or 72 hours of treatment, cells were incubated with 1 mg/ml of MTT for 4 hours at 37°C in a CO2 incubator. Mitochondrial reduction of MTT to formazan was determined. Amount of formazan was measured by absorbance at 570 nm with reference wavelength at 620 nm. Graphs were plotted as mean±standard deviation of three independent experiments. B) Live-Dead assays were performed after 24, 48, 72 and 96 hrs post MPTQ treatment as described in figure 1 and compared with their corresponding controls. Images were acquired and displayed with identical settings. C) Percentage of live cells and dead cells were calculated as described in figure 1 and were plotted as histograms of mean±standard deviation of three independent experiments. p value displayed for each treatment time was calculated by comparing with control sample using Student’s t-test. p value ≤5×10^−2^ is considered significant.

Since MPTQ had moderate cell toxicity on neuro 2a cells at 24 hrs post treatment, we examined whether its cytotoxicity effect continued beyond 24 hrs post treatment. Neuro 2a cells were treated with 30 µM of MPTQ and cytotoxicity was studied at 24, 48, 72 and 96 hrs post treatment. We utilized both MTT and Live-Dead assays to monitor the progression of cytotoxicity. MTT assay is a gross indicator of the presence of live cells that convert MTT to formazan products. However, it may not be an ideal experiment for absolute quantification of live cells in experimental culture conditions. Live-Dead assay provides robust platform to visualize even a single live cell in a particular experimental culture. Analysis of MTT assay demonstrated significant time dependent increased cytotoxicity in MPTQ treated neuro 2a cells. The amount of formazan product almost reached zero (0.044±0.035) by 72 hrs while the untreated cells continued to grow during this period (Figure 2A). Fluorescent images from Live-Dead analysis also showed significant time-dependent cell death in MPTQ (30 µM) treated neuro 2a cells (Figure 2B) than control cells. After 96 hrs of MPTQ treatment, we were able to detect only 6 live cells out of 36 random images across 3 independent experiments, where as in corresponding controls we found 5844 live cells. The percentage of live cell population in control groups show more than 90% cells are live till 72 hrs but decreased to 72% by 96 hrs in culture, possibly because of depletion of nutrients in culture media. In contrast, percentage of live cells in MPTQ treated groups showed 65.6, 10.6, 1.53 and only 0.19% after 24, 48, 72 and 96 hrs of treatment respectively. This change was statistically significant by one way ANOVA, F = 48.896 and p<0.001 and as well as by two tailed unpaired Student’s t-test (Figure 2C). Thus, MPTQ retains its cytotoxic property over a long period of time which resulted in a strong inhibition of neuro 2a neuroblastoma cell proliferation.

### MPTQ Treatment also Induces Strong Cytotoxic Effect on Human SH-SY5Y Neuroblastoma Cells

**Figure 3:**
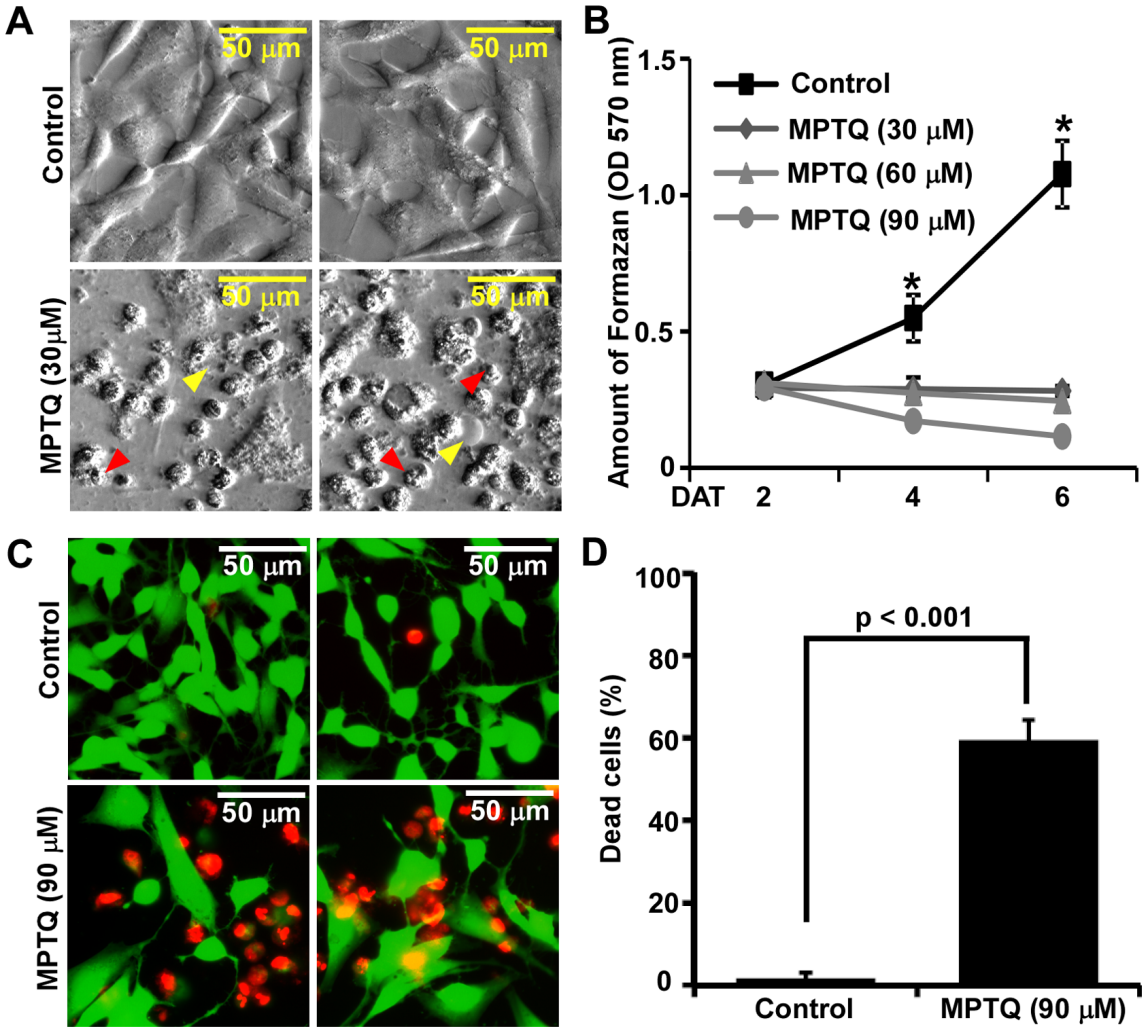
Cytotoxic and antiproliferative effect of MPTQ on human SH-SY5Y neuroblastoma cells. A) Bright field images after 6 days of 30 µM MPTQ treated cells exhibit gross morphological disintegration compared to untreated cells. SH-SY5Y cells demonstrate plasma membrane blebbing with cytoplasmic oozing (yellow arrow head) and irregular nuclear compaction and fragmentation (red arrow head) only in MPTQ treated cells but not in normal cells. B) Antiproliferative effect of MPTQ on SH-SY5Y cells by MTT assay. Cells were treated with 30, 60 or 90 µM of MPTQ or with equal amount of DMSO (control) for 2, 4 and 6 days. After each time point, MTT assay was performed as described earlier. Graphs were plotted as mean±standard deviation of three independent isolates. * indicates the differences between untreated and treated samples are statistically significant (p<0.05; Student’s t-test). C) Live-Dead assay on MPTQ (90 µM) treated SH-SY5Y cells show increased number of dead cells after 4 days over untreated cells. D) Number of dead cells were counted from three random fields and from three separate experiments and plotted as histogram (mean±standard deviation). p-value ≤0.05 represents statistical significance between the mean of untreated and MPTQ treated SH-SY5Y cells. DAT = days after treatment.

To extend our study on the cytotoxic effect of MPTQ on human neuroblastoma, we modelled SH-SY5Y, a slow growing human neuroblastoma cell line. Bright field images show healthy cells in untreated cells where as MPTQ treated cells showed extensive cell shrinkage, membrane blebbing, chromosomal condensation, nuclear fragmentation and cell death (Figure 3A). Analysis of MTT assay demonstrated dose-dependent anti-proliferative effect of MPTQ on SH-SY5Y cells. Untreated cells continue to proliferate till 6 days in culture whereas SH-SY5Y cell proliferation was strongly inhibited by all tested doses of MPTQ. By fourth day post treatment, significant difference between untreated and all the doses of MPTQ treated SH-SY5Y cells were observed. Moreover 90 µM of MPTQ treatment was found to be most efficient and was able to inhibit the proliferation of SH-SY5Y cells by approximately 90% over untreated cells (Figure 3B). To support our results further, we employed Live-Dead assay. Untreated cells showed merely 1.65% dead cells after 4 days of culture whereas, 90 µM of MPTQ treated SH-SY5Y cells exhibited as high as 59.53% dead cells after 4 days (Figure 3C and D). The difference between untreated and treated cells is statistically significant by Student’s t-test (p<0.001). Taken together our results strongly suggest the cytotoxicity effect of MPTQ not only on a fast growing mouse neuro 2a neuroblastoma cell line but also on human SH-SY5Y neuroblastoma cell line. Since neuro 2a cells were more responsive to MPTQ treatment, these cells were utilized in subsequent studies on molecular mechanisms of MPTQ-mediated neuroblastoma cell death.

### MPTQ Induces DNA Fragmentation in a Dose Dependent Manner in Neuro 2a Neuroblastoma Cells

**Figure 4:**
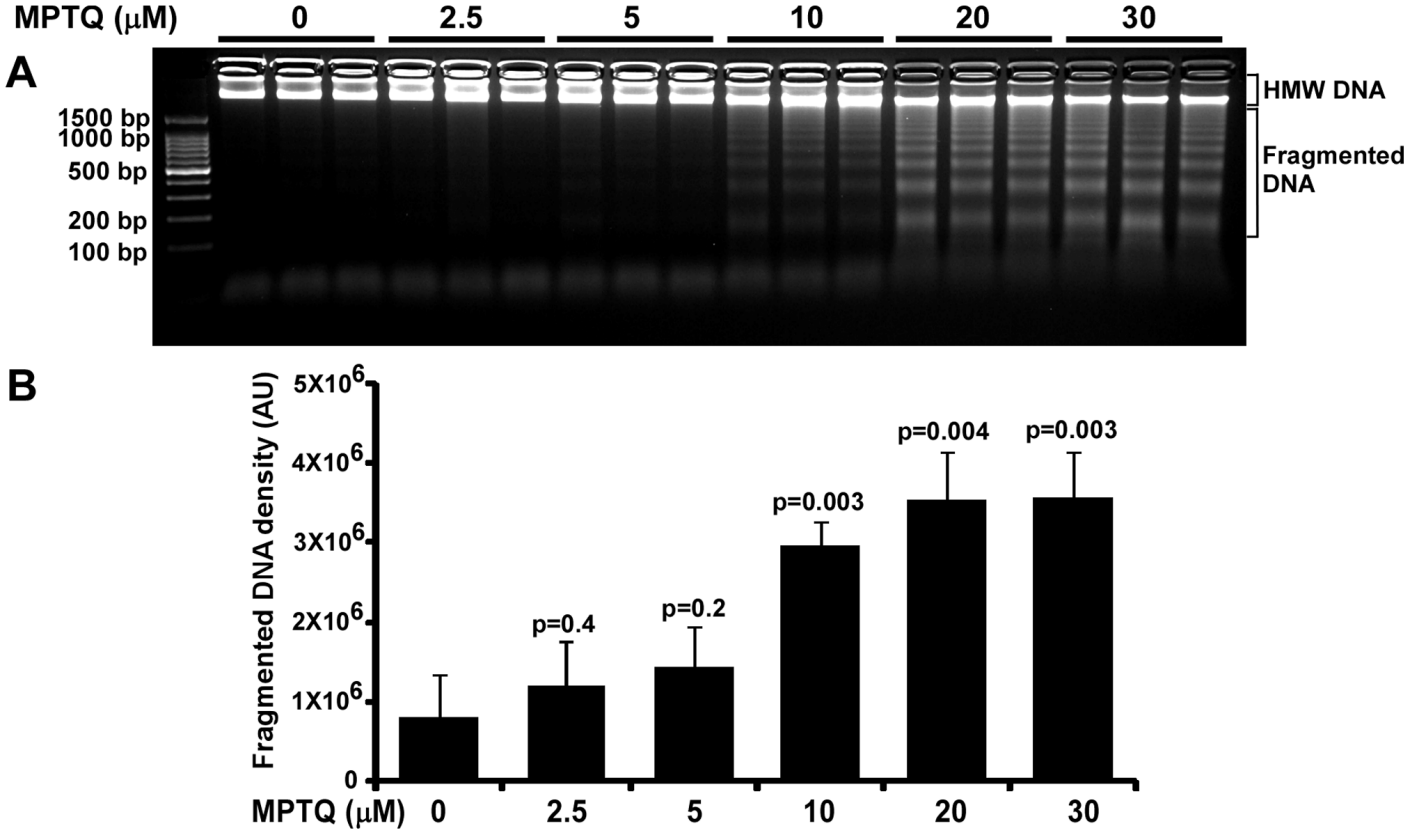
MPTQ induces nuclear DNA fragments of oligonucleosomal size in neuro 2a cells. A) Agarose gel electrophoresis of genomic DNA isolated from untreated and 2.5, 5, 10, 20 or 30 µM MPTQ treated cells for 48 hours indicated dose-dependent increased DNA fragmentation. B) Intensity of fragmented DNA was calculated and mean with standard deviation was plotted. Data represents three independent experiments. p value displayed for each duration of treatment was calculated by comparing with control sample using Student’s t-test. p value ≤5×10^−2^ is considered significant. AU = Arbitrary Units.

The DNA intercalation property of MPTQ [18] might interfere with DNA replication and induce nuclear DNA insults including DNA double strand breaks (DSBs). Presence of DSBs in the form of oligonucleosomal DNA ladder is an indicator of apoptosis [31]. To examine this in MPTQ treated neuro 2a cells, we utilized DNA fragmentation assay. Total genomic DNA was isolated from neuroblastoma cells after 48 hrs of treatment with 2.5, 5, 10, 20 or 30 µM of MPTQ. Agarose gel electrophoresis of genomic DNA showed DNA ladders of oligonucleosomal size (integer multiples of 180 bp) from 10 µM onwards and became prominent at 20 and 30 µM (Figure 4A). The densitometric analysis of fragmented DNA showed significant dose-dependent increased DNA fragmentation in MPTQ treated neuro 2a cells over corresponding controls (One way ANOVA, F = 17.142, p<0.001). In addition, two tailed unpaired Student’s t-test also indicated significant difference between untreated controls and from 10 µM onwards of MPTQ treatment (Figure. 4B). Taken together, our results indicate the possible involvement of apoptosis in MPTQ-mediated cell toxicity in neuro 2a neuroblastoma cells.

### MPTQ-mediated Formation of Nuclear DNA Fragments were Positive for TUNEL, a Marker of Apoptosis

**Figure 5:**
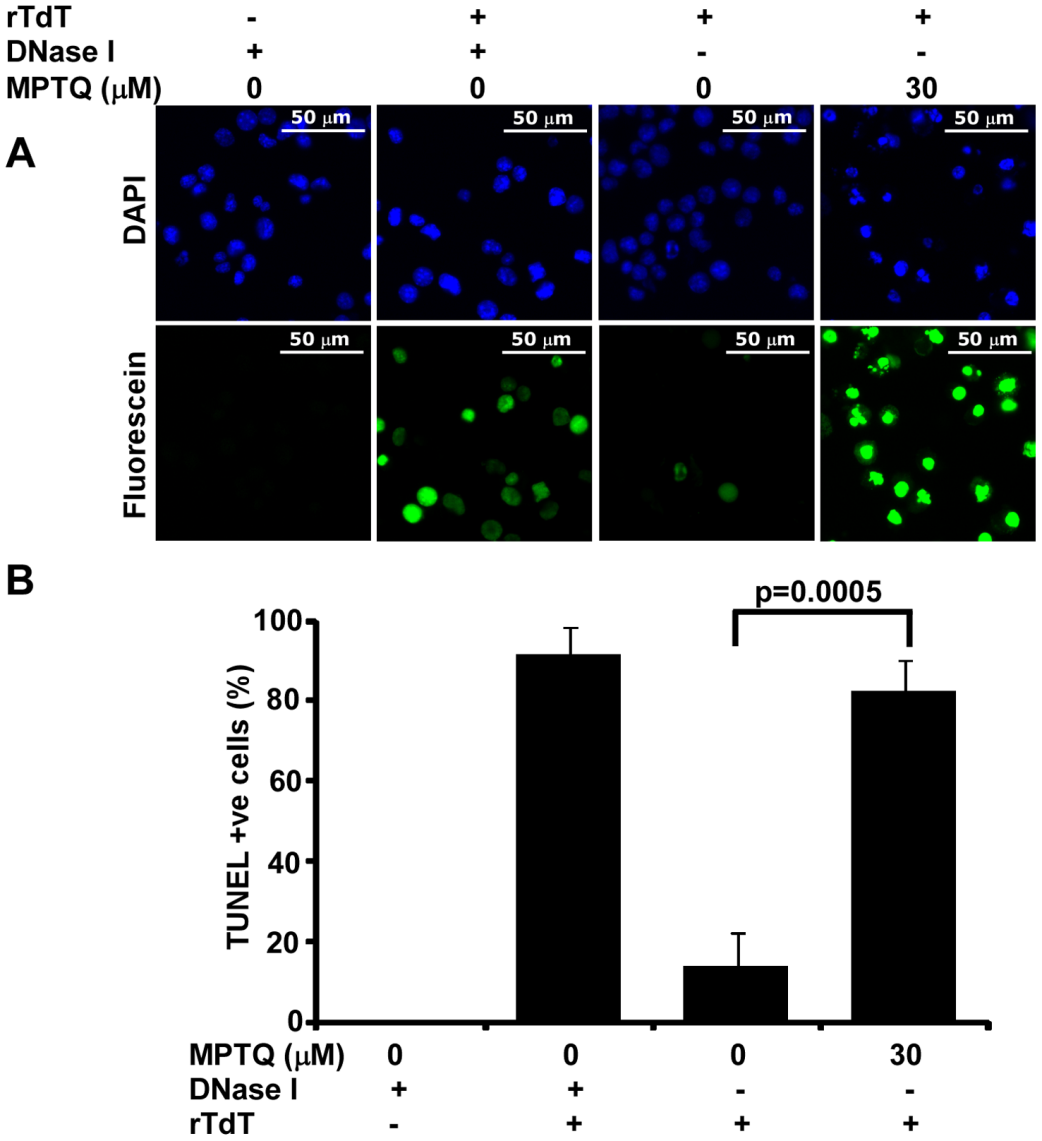
MPTQ induced nuclear DNA breaks in neuro 2a cells are positive for TUNEL staining, an indicator of apoptosis. Neuro 2a cells were cultured and treated with 30 µM of MPTQ for 48 hrs. Control cells were treated with equal amount of DMSO. A) Fluorescent images of DAPI and fluorescein-12-dUTP were captured from multiple random fields using multi-dimension acquisition module of MetaMorph using identical settings and images were displayed with equal pixel intensity. Images display increased TUNEL positive neuro 2a cells in MPTQ treated cells than control cells B) Number of TUNEL positive cells were calculated using multi-cell scoring module of MetaMorph software and mean of three independent experiments are presented as histograms with standard deviation as error bars. Results indicated more than 80% cells were positive for TUNEL in MPTQ treated cells where as only 13% in control cells. p value was calculated by Student’s t-test and displayed. p≤0.05 is considered statistically significant.

Apoptotic DNA fragmentation is characterized by blunt end, 3′ and 5′ overhangs, out of which 3′ overhangs are exclusively found in apoptotic cells but not in necrotic cells. Terminal deoxynucleotidyl transferase (TdT), key enzyme in TUNEL assay has higher preference to label DNA fragments with 3′ overhangs generated during apoptosis [32], [33]. We therefore utilized TUNEL assay to detect cells undergoing apoptosis. Neuro 2a neuroblastoma cells were treated with 30 µM of MPTQ and cells were harvested after 48 hrs of treatment. DMSO treated samples served as experimental control. Fluorescence images from negative and positive controls exhibited results as per our expectation suggesting the methods and reagents were working satisfactory. In experimental samples, majority of MPTQ treated neuro 2a cells were positive for TUNEL staining whereas most of the cells were negative for TUNEL staining in untreated cells (Figure 5A). Cell scoring analysis further demonstrated that, approximately 82% of cells were positive for TUNEL staining in MPTQ treated neuro 2a cells but only 13% in corresponding controls (Figure 5B) and the difference between these groups are significant by Student’s t-test (p = 0.0005). Hence, our results strongly suggest the activation of cellular apoptotic machineries in MPTQ-mediated neuro 2a cell death.

### Activation of ATM as a Marker of DNA Double Strand Breaks in MPTQ Treated Neuro 2a Neuroblastoma Cells

**Figure 6:**
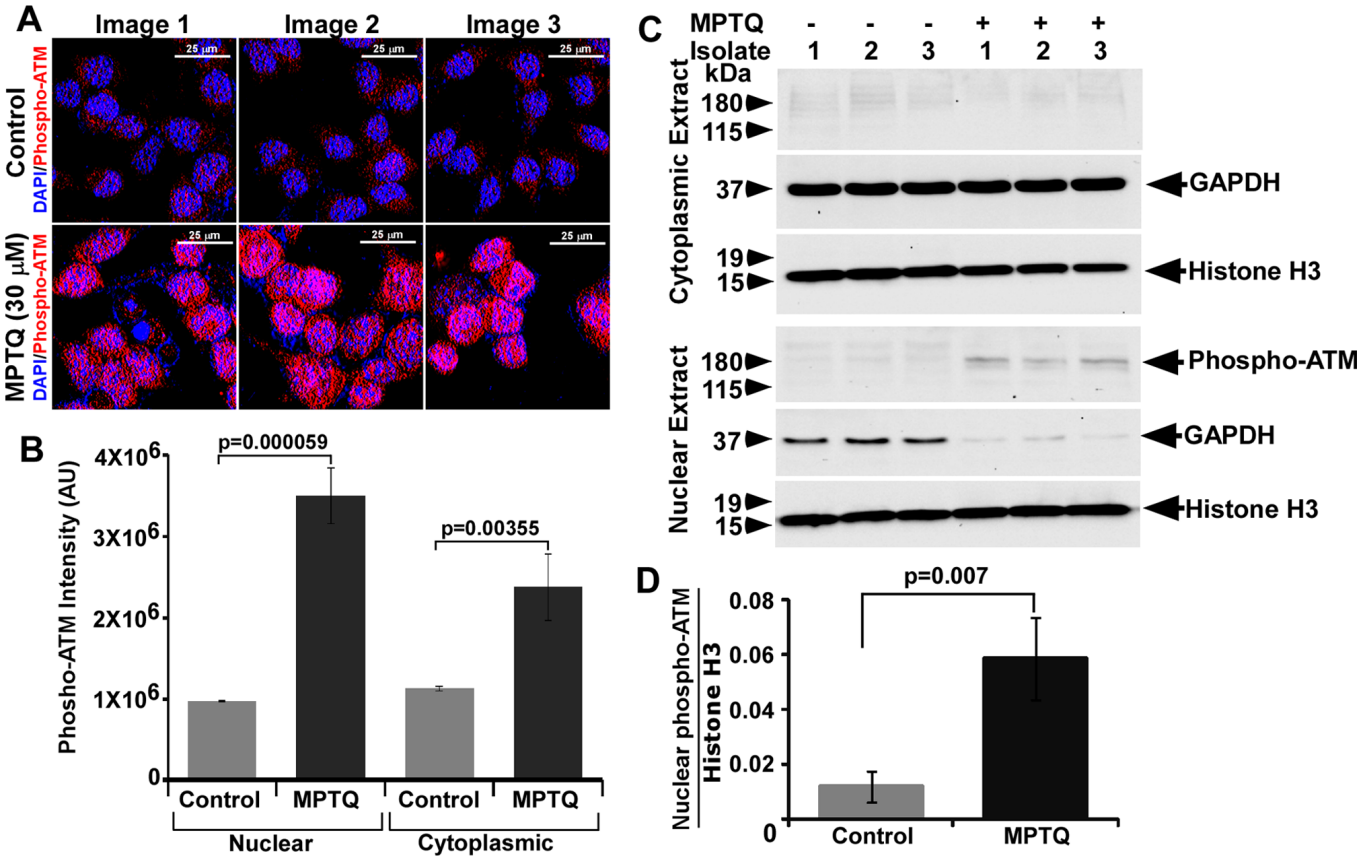
MPTQ-mediated neuro 2a neuroblastoma cell death is associated with increased phosphorylation of ATM. A) 30 µM of MPTQ treated or untreated cells were fixed after 24 hours of treatment and immunolabelled with an antibody specific for phosphorylated ATM (ser1983). Detection was done using Alexa fluor 594 labelled secondary antibodies. Nuclei were stained by DAPI. Z-stack images were captured from multiple random fields, processed and displayed as described in methods. B) Intensity of phospho-ATM was measured in cytoplasmic and nuclear compartment. Histograms represent mean±standard deviation of three independent images from two independent experiments. C) Western blot analysis of phosphorylated ATM on cytosolic and nuclear fraction of MPTQ treated or untreated neuro 2a cells. Blots were also immunoblotted with anti-GAPDH and anti histone H3 antibodies for normalization. The results indicate increased phosphorylated ATM level in nuclear compartment of MPTQ treated cells than untreated cells. D) Densitometric analysis of phosphorylated ATM bands was performed and the values are plotted as mean±standard deviation of three independent isolates. Statistical analysis was made by Student’s t-test and p value is displayed. p value ≤0.05 is considered significant.

Our earlier results showed nuclear DNA fragmentation and TUNEL positive cells in MPTQ treated neuro 2a Cells. Accumulation of fragmented DNA is a clear indicator of DNA double-strand breaks (DSBs) which are potent biological genotoxic lesions [34], [35]. The key regulator of the DNA DSBs response is a nuclear protein coded by Ataxia-Telangiectasia mutated (ATM) gene [36]. It is a serine/threonine protein kinase. DNA DSBs induce conformational changes in ATM and stimulate autophosphorylation of ATM [37], [38], [39]. In order to study the mechanism related to apoptosis and genotoxic effect of MPTQ on neuro 2a neuroblastoma cells, we utilized an antibody specific for phosphorylated ATM that recognizes activated mouse ATM (phophorylated at Ser1983). Our immunocytochemistry results demonstrated increased level of activated ATM in MPTQ treated neuro 2a cells than their corresponding controls (Figure 6A). The intensity of phosphorylated ATM in nuclear compartment was found to be approximately 3-fold more in MPTQ treated than untreated neuro 2a cells (Figure 6B). We also found increased phosphorylated ATM in cytoplasm of MPTQ treated than untreated neuro 2a cells (Figure 6B). To validate this result further, western blot analysis of phosphorylated ATM was performed on cytosolic and nuclear fractions of MPTQ treated and untreated neuro 2a cells. Our result demonstrated decrease in GAPDH level in nuclear fractions than cytosolic fractions which suggests the enrichment of nuclei in our preparation. Results from these nuclear extracts demonstrated increased phosphorylated ATM level in all the isolates of MPTQ treated cells than corresponding controls (Figure 6C). However, unlike our immunocytochemical result, phosphorylated ATM was not observed in the cytosolic fraction of MPTQ treated and untreated isolates (Figure 6C). Such difference between immunocytochemistry and western blot could be due to low level of phosphorylated ATM protein in cytosolic fractions (which are roughly 10 times diluted than nuclear extract) or due to the difference in the efficiency of antibody-antigen interaction in these two assay systems. Densitometric analysis of phosphorylated ATM band in nuclear fractions demonstrated approximately 4.8-fold more in MPTQ treated cells than untreated isolates and the difference is statistically significant (Student’s t-test; p = 0.007) (Figure 6D). Taken together, activation of ATM suggests MPTQ as a genotoxic agent which might have induced DNA DSBs in neuro 2a neuroblastoma cells and might have engaged cells to activate other factors associated with apoptosis.

### Activation of p53 is Associated with MPTQ-mediated Apoptosis in Neuro 2a Neuroblastoma Cells

**Figure 7:**
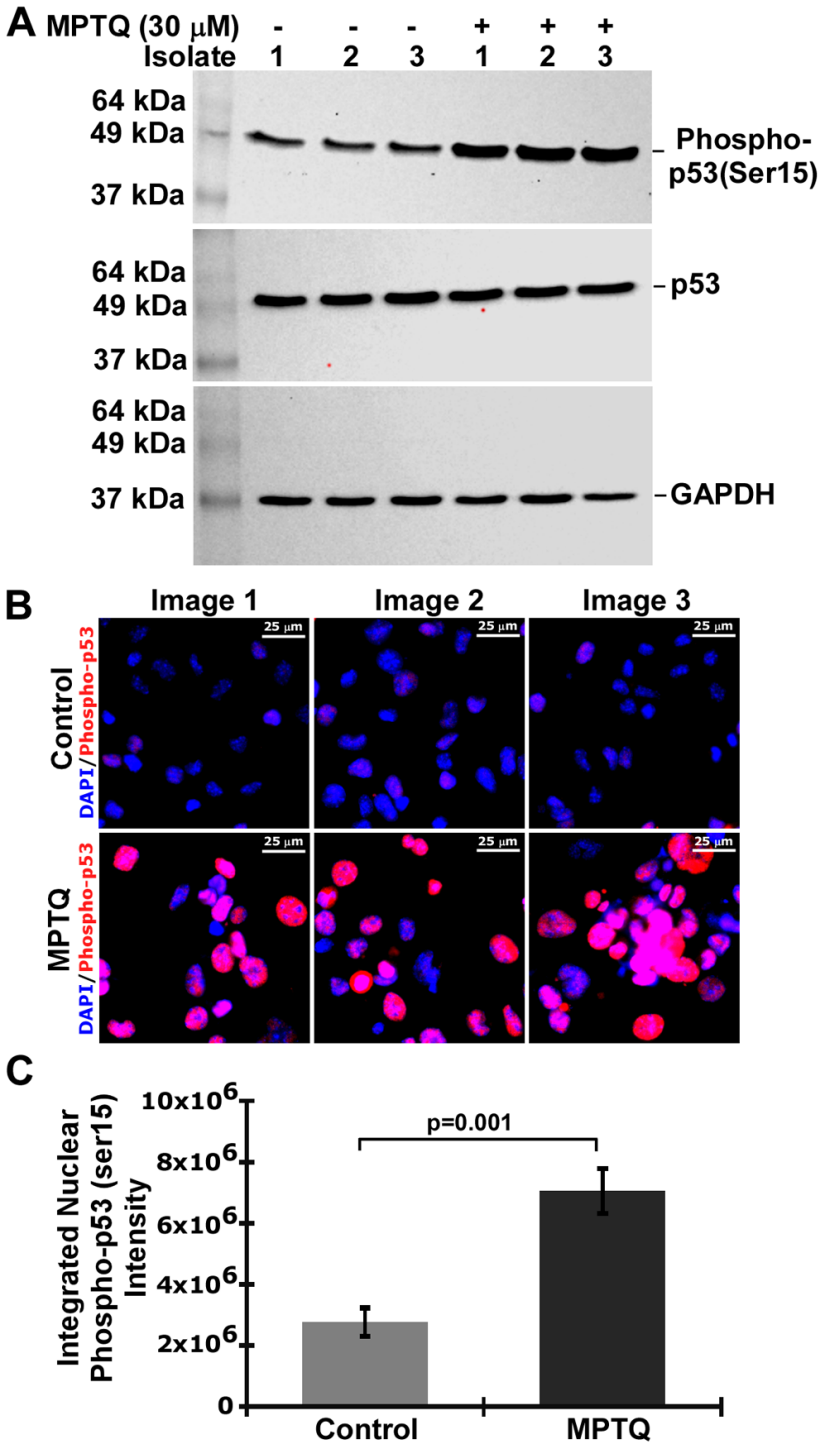
MPTQ-mediated cell death is associated with increased phosphorylation of p53 at Ser15. A) Western blot analysis of phospho-p53 (Ser15), p53 and GAPDH. Neuro 2a cells were either treated with 30 µM of MPTQ or DMSO alone for 24 hours. Three independent isolates were obtained and 60 µg of total proteins were size fractionated in 12% SDS-PAGE and western blotted either with anti-phospho-p53 (ser15) or with anti-p53 antibody. The blots were stripped and hybridized with anti-GAPDH antibody to normalize any loading difference. B) Immunocytochemistry of phopho-p53 (Ser15). Images represent three independent experiments C) Nuclear phospho-p53 (Ser15) intensity was measured as described in figure 6. Histograms represent mean integrated nuclear phopho-p53 (Ser15) intensity±SD of three independent experiments. p value calculated by Student’s t-test is displayed which indicates significant increased phosphorylation of p53 at Ser15 in MPTQ treated neuroblastoma cells.

One of the downstream targets of activated ATM is p53 protein, the guardian of the genome [40]. Activated ATM can phosphorylate p53 at Ser15 [41], [42] and Ser20 [43], [44]. In addition, p53 is a key regulator to various cellular stress responses, specifically those that induce DNA damage. Since MPTQ is a DNA intercalating agent and has activated ATM in our study, we therefore examined the induction of p53 and activation of p53 by its phosphorylation at Ser15 and 20. We utilized both western blot and immunocytochemical analysis. Western blot analysis of neuro 2a cells lysates obtained after 24 hrs of MPTQ (30 µM) treatment demonstrated increased phosphorylation of p53 at Ser15 than untreated cells. However, no significant change in the amount of total p53 and GAPDH was observed between treated and control cell lysates (Figure 7A). A more robust data was also obtained when cells were immunolabelled *in situ* with an anti-phospho-p53 (Ser15) antibody. Increased level of nuclear phosphorylated p53 (Ser15) was observed in MPTQ treated neuro 2a cells than control cells (Figure 7B). The nuclear intensity of phosphorylated p53 (Ser15) was measured and results show approximately 2.55-fold more of phosphorylated p53 (Ser15) in MPTQ treated neuro 2a cells than their corresponding controls (Figure 7C) and is statistically significant by Student’s t-test (t = –8.562 and p = 0.001). Furthermore, nuclear phosphorylated p53 (Ser20) level was also more in MPTQ treated neuro 2a cells than untreated neuro 2a cells by immunocytochemical analysis (data not shown). Thus, activation of p53 correlates with activation of ATM and suggests MPTQ as a potent genotoxic agent, which might be activating apoptotic pathways in neuro 2a cell deaths.

### MPTQ-mediated Neuro 2a Neuroblastoma Cell Death is Associated with Bax Induction

**Figure 8:**
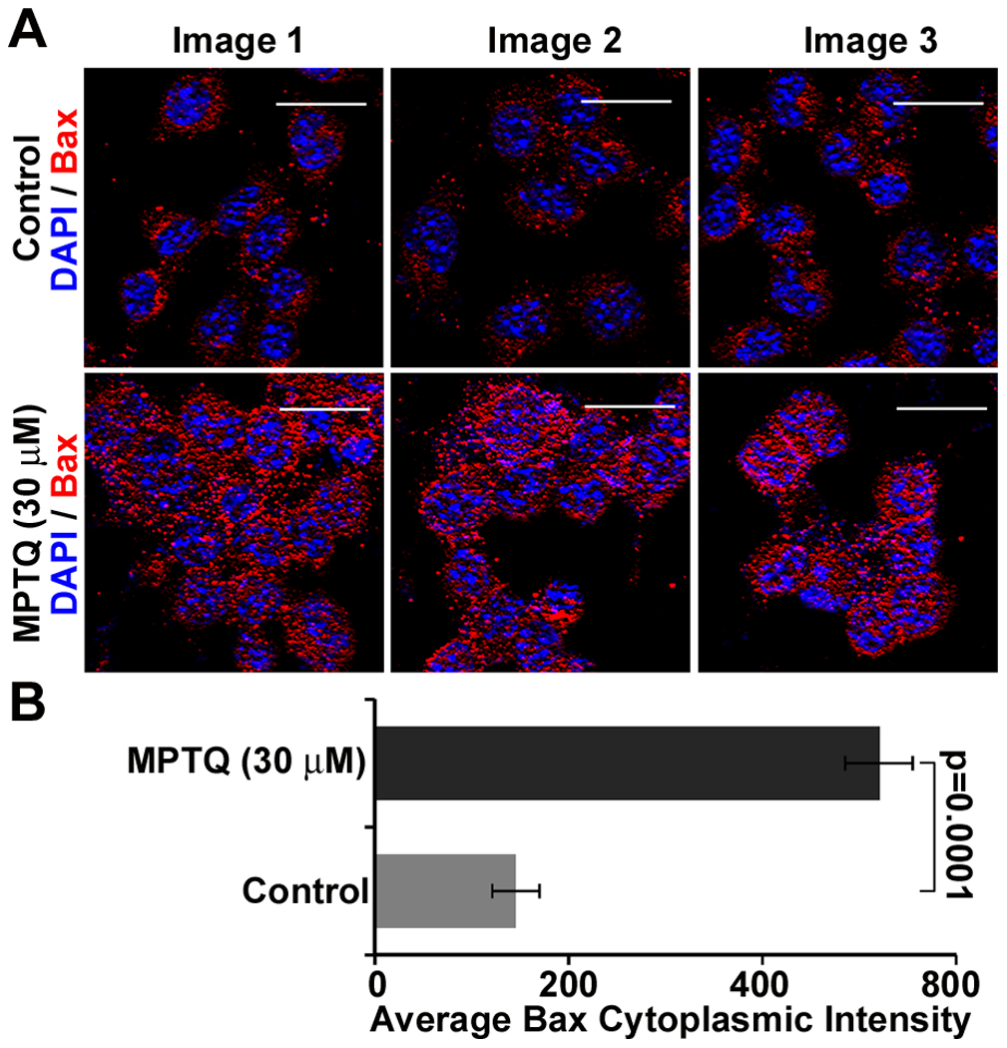
MPTQ treatment increases Bax protein expression and redistribution in neuro 2a neuroblastoma cells. A) Neuro 2a cells were cultured and treated with 30 µM of MPTQ for 24 hours followed by immunocytochemistry for Bax expression by an anti-Bax antibody. Detection was done using Alexa 594 labelled secondary antibody. Nuclei were stained with DAPI. B) Cytoplasmic level of Bax immunosignal was obtained using multi-cell scoring module of MetaMorph software and mean of three random images were displayed as histograms. Error bar indicates standard deviation. p value was calculated by Student t-test and is displayed which strongly indicates the overexpression of Bax protein in MPTQ treated neuro 2a cells.

The requirement of Bax for p53-mediated apoptosis [45] and enhancement of p53-mediated transcriptional activity by the phosphorylation of p53 at Ser15 has been reported [42]. Bax, the first member of Bcl-2 family of pro-apoptotic proteins is known to be induced by p53 [46]. It has been shown earlier that increased expression of Bax enhances cell death by various apoptotic stimuli [47]. Since p53 is activated in our study, we examined the possible involvement of Bax in our study. Fluorescent images from immunocytochemistry demonstrated increased Bax-immunoreactivity in MPTQ treated neuro 2a cells. In untreated cells a diffused Bax signal was observed but more of punctuated Bax signal was observed in MPTQ treated neuro 2a cells (Figure 8A). Intensity measurement analysis show 3.5-fold more Bax immunosignal in the cytoplasm of MPTQ treated neuro 2a cells than corresponding controls (Figure 8B). Collectively, induction of Bax protein for the first time suggests the possible involvement of mitochondrial apoptosis pathway in MPTQ-mediated cell death in neuro 2a neuroblastoma cells.

### MPTQ-mediated Neuro 2a cell Death Activates Caspases Involved in Intrinsic Apoptotic Pathway

**Figure 9:**
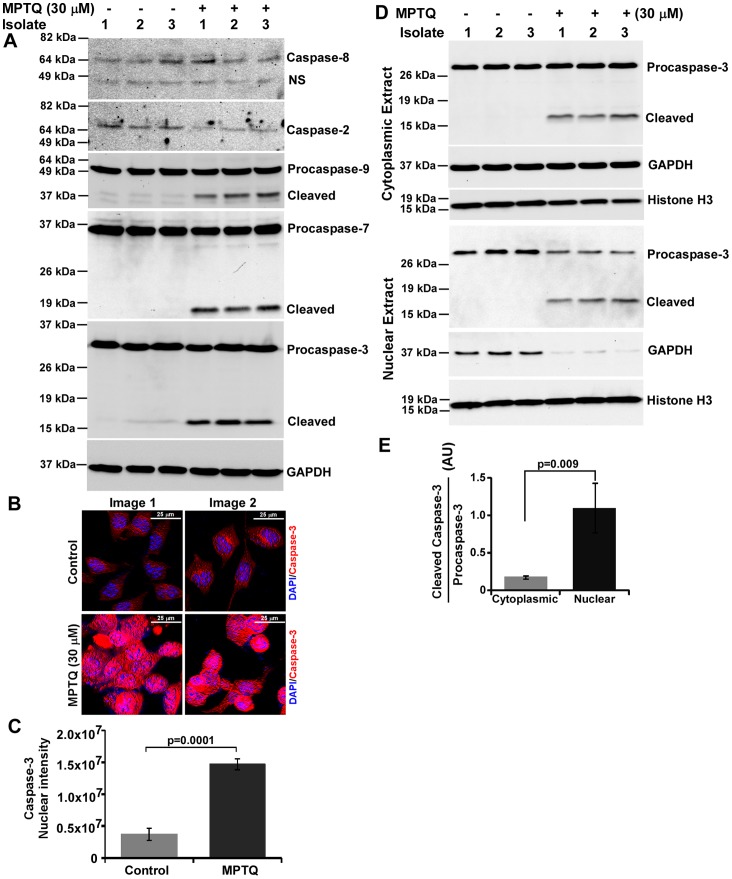
MPTQ-mediated cell death is associated with activation of caspases of intrinsic apoptosis pathway but not of extrinsic pathway. A) Neuro 2a cells were cultured and treated with 30 µM of MPTQ for 24 hours and lysates were prepared. 60 µg of total proteins were resolved in 12% SDS-PAGE and immunoblotted with anti-caspase-8 or anti-caspase-2 or anti-caspase-9 or anti-caspase-3 or anti-caspase-7 antibody. Blots were stripped and immunoblotted with anti-GAPDH antibody. The results clearly indicate the activation of caspase-9, -3 and-7 but not caspase-8 and -2 in MPTQ treated cells. B) Immunocytochemistry of caspase-3 protein was performed as described earlier. Increased caspase-3 level was observed in the nucleus of MPTQ treated neuro 2a cells but not in control cells. C) Nuclear level of caspase-3 immunosignal was obtained using multi-cell scoring module and mean of three random images of two independent experiments were displayed as histograms. Error bar indicates standard deviation. D) Western blot analysis of cleaved caspase-3 level in cytosolic and nuclear fraction of MPTQ treated or untreated neuro 2a cells. Blots were also immunoblotted with anti-GAPDH and anti-histone H3 antibodies for normalization. E) Densitometric analysis of procaspase-3 and cleaved caspase-3 bands were made from cytosolic as well as from nuclear fractions. Cleaved caspase-3 to procaspase-3 ratio was obtained. Mean and standard deviation from three independent isolates were obtained and plotted as histograms. p value was calculated by Student’s t-test and is displayed which indicates significant increased mobilization of cleaved caspase-3 from cytoplasm to nucleus in MPTQ treated neuro 2a cells.

In p53-Bax activation pathway, mitochondrial outer membrane permeabilization by Bax triggers the initiation of intrinsic apoptosis pathway. Interaction of caspase-9 with Apaf-1, in the presence of cytochrome-c released from mitochondrial intermembrane space activates apoptosome that further activate downstream caspases. During this process, casapse-9 is autoprocessed to finish the apoptosome acitivity. Thus, proteolytic activation of caspase-9 (an initiator caspase of intrinsic apoptotic pathway) acts as an indicator of the initiation of intrinsic apoptosis pathway as well as permeabilization of mitochondria [48], [49]. Activation of p53 also has the ability to activate extrinsic apoptotic pathway in which, proteolytic activation of caspase-8 serves as a key marker. Nothing is known about these pathways in MPTQ-mediated cell death in any neuroblastoma cells. To study both the apoptotic pathway, we examined activation of caspases related to extrinsic as well as intrinsic apoptotic pathways in MPTQ-mediated cell death in neuro 2a neuroblastoma cells. Western blot results strongly demonstrated the activation of caspase-9 but not caspase-8 in MPTQ treated neuro 2a cells (Figure 9A), suggesting the activation of only intrinsic apoptotic pathway. Furthermore, processed product of caspase-2 was not observed in our study indicating caspase-9 activation as the main initiation event in MPTQ-mediated activation of intrinsic apoptotic pathway in neuro 2a cells. Downstream targets of caspase-9 are caspase-3 and caspase-7. Both caspase-3 and caspase-7 were activated in MPTQ treated neuro 2a cells (Figure 9A). Activated caspase-3 has been known to have targets in nuclear compartment for the initiation of DNA damage [50], [51] suggesting its nuclear localization. To study the localization of caspase-3, we employed immunocytochemistry. Increased caspase-3 signals were found in MPTQ treated neuro 2a cells than untreated cells (Figure 9B). Fluorescent intensity measurement demonstrated caspase-3 nuclear signal is approximately 3-fold more in MPTQ treated neuro 2a cells than controls and is statistically significant (p = 0.0001) (Figure 9C). Although cytoplasmic signals were also more in treated cells but the difference in the mean between MPTQ treated and untreated cells is not significant (p = 0.08; data not shown). To validate the localization of cleaved caspase-3 in MPTQ treated neuro 2a cells, western blot analysis were performed separately on cytosolic and nuclear fraction of untreated and treated cells. Similar to our previous result, no cleaved caspase-3 products were seen in both the fraction of untreated cells. However, cleaved caspase-3 were clearly seen in both the fractions of MPTQ treated cells (Figure 9D). Moreover, densitometric analysis of cleaved caspase-3 and procaspase-3 band indicates approximately 6-fold increased cleaved caspase-3 in the nuclear fraction than cytosolic fraction of MPTQ-treated cells (Figure 9E) indicating the translocation of cleaved caspase-3 into the nucleus of MPTQ-mediated cytotoxicity in neuro 2a cells and its role in the nucleus of apoptotic cells. Taken together, our results clearly suggest the involvement of intrinsic but not extrinsic apoptotic pathway in MPTQ-mediated neuro 2a cell death.

### MPTQ Inactivates PARP in Neuro 2a Neuroblastoma Cells

**Figure 10:**
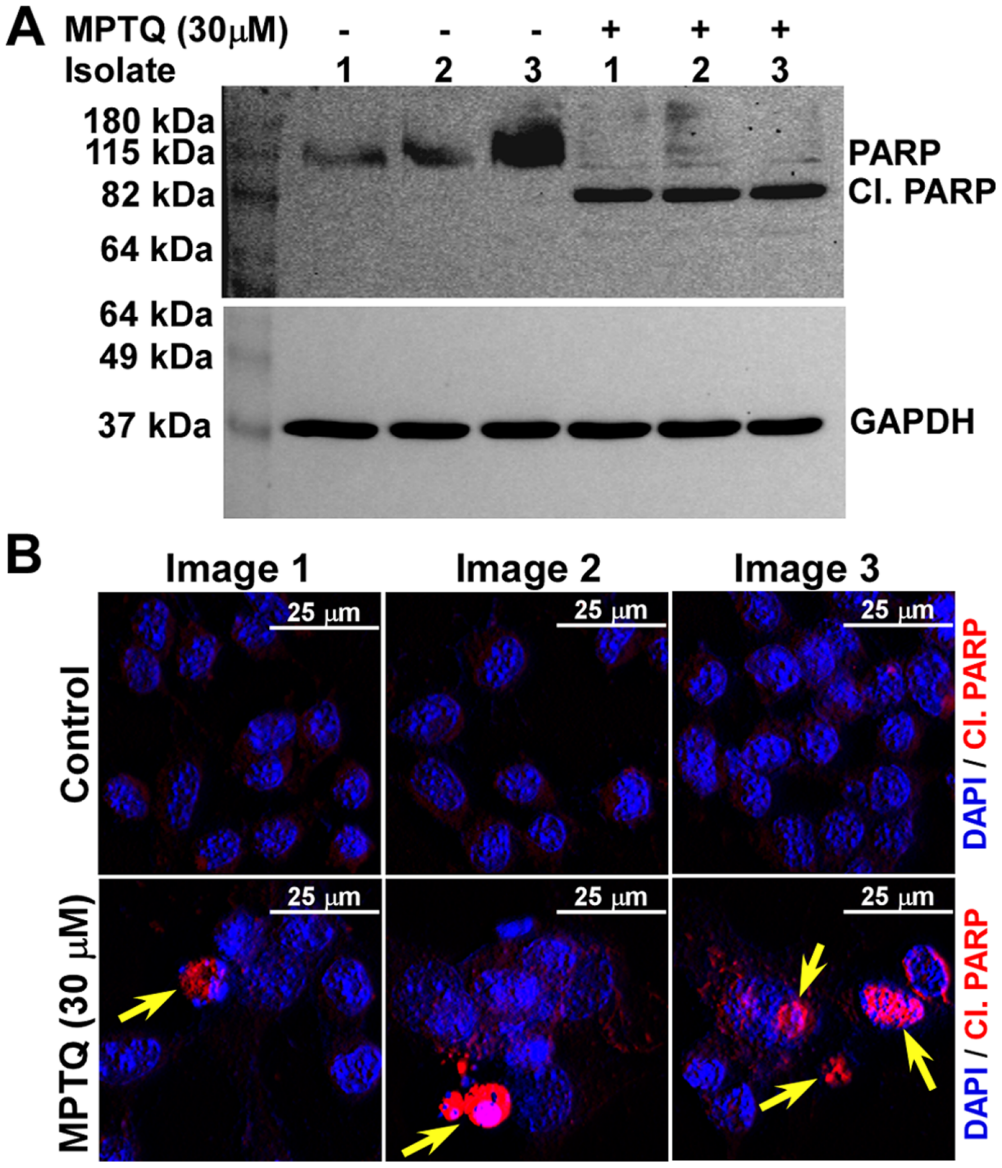
Increased proteolysis of PARP in MPTQ treated neuro 2a neuroblastoma cells. A) Neuro 2a cells were cultured and treated with 30 µM of MPTQ for 24 hours and lysates were prepared from three independent treatments. 60 µg of total protein were size fractionated in 12% SDS-polyacrylamide gel and immunoblotted with anti-PARP antibody that detects both full length PARP and cleaved PARP. Cleaved PARP is seen only in MPTQ treated N2a cells but not at all in control cells indicating the proteolysis of PARP, a hallmark feature in apoptotic cells. B) Immunocytochemistry of PARP using an antibody specific for cleaved PARP. Detection was done using Alexa fluor 594 labelled secondary antibodies. Nuclei were stained with DAPI. Nuclei with cleaved PARP were seen only in MPTQ treated cells. The figure represents at least three independent experiments.

Poly (ADP Ribose) polymerase (PARP) is a 116 kDa chromatin-associated protein that binds with DNA strand breaks and catalyzes long branched polyADP-ribose on many nuclear protein and on itself using NAD+ [52]. PARP is a nuclear target of cleaved caspase-3 and caspase-7 and proteolytic cleavage of PARP is considered to be a hallmark feature of apoptosis [53], [54], [55], [56]. PARP is cleaved by these caspases between Asp214 and Gly215 residues to produce 24 kDa and 89 kDa fragments with the loss of its catalytic activity [55], [56]. Since increased proteolytic activation of caspase-3 and caspase-7 was found in MPTQ treated neuro 2a cells, we studied the proteolysis of PARP by western blot and immunocytochemical analysis. Western blot analysis demonstrated the presence of only full length PARP protein in untreated cells but a strong proteolytic 89 kDa PARP fragment and weak 116 kDa full length PARP protein in all the isolates of MPTQ treated cells (Figure 10A). In continuation, we utilized an antibody specific for cleaved PARP in our immunocytochemical studies. Images from immunocytochemical analysis demonstrate the presence of cleaved PARP positive cells only in MPTQ treated neuro 2a cells but not at all in control cells (Figure 10B). Thus, proteolysis of PARP correlates with the increased nuclear DNA fragmentation in MPTQ treated neuro 2a cells and strongly suggests the involvement of apoptosis.

### MPTQ Treatment Increases Nuclear Localization of AIF in Neuro 2a Neuroblastoma Cells

**Figure 11:**
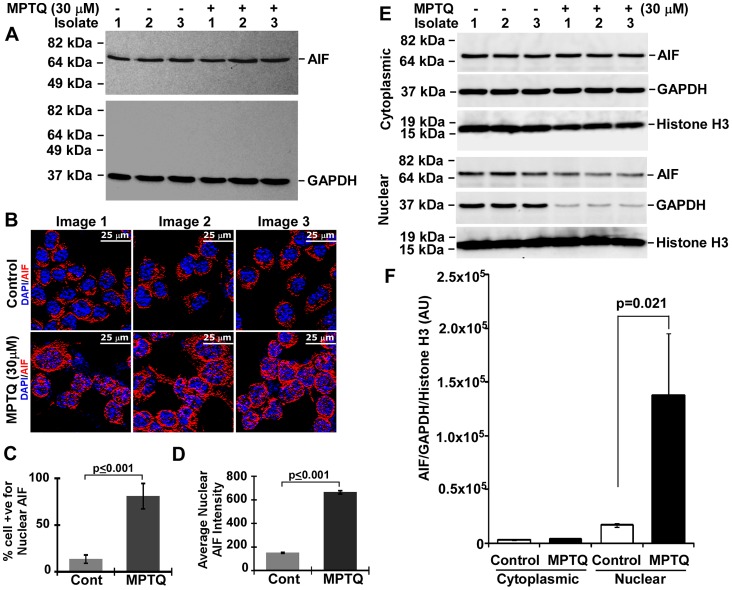
MPTQ engages caspase independent intrinsic apoptosis pathway through AIF nuclear translocation. A) Western blotting was performed on three untreated and three MPTQ (30 µM) treated neuro 2a cell lysates using an antibody specific for AIF showing no change in AIF expression between treatments. B) Immunocytochemistry of AIF was used to monitor AIF cellular localization. Detection was done using Alexa 594 labelled secondary antibody. Nuclei were stained with DAPI. Analysis of images indicates percentage of cells positive for nuclear AIF is significantly more in MPTQ treated cells than control cell (C) and the level of nuclear AIF level is also significantly more in MPTQ treated cells than control neuro 2a cells (D). E) Western blot analysis of AIF, GAPDH and histone H3 on 3 independent sets of cytoplasmic and nuclear fraction of neuro 2a cells. F) Densitometric analysis indicated approximately 8-fold increased AIF level in nuclear compartments of MPTQ treated cells than untreated cells after normalization with GAPDH and histone H3. Statistical analysis was made by Student’s t-test and p values are displayed. p value ≤0.05 is considered significant.

Mitochondria-mediated apoptosis not only occurs through caspase-mediated activation of intrinsic apoptotic pathway but can also occur through an alternative mitochondrial route, independent of caspases [57]. During mitochondrial outer membrane permeabilization several mitochondrial intermembrane proteins are released into the cytoplasm and activates pro-apoptotic stimulus independent of caspases. One such protein is apoptosis inducing factor (AIF). Upon genotoxic stimulus, AIF translocates from mitochondria to cytoplasm and nucleus, where it induces large scale DNA degradation [58]. To study the possible involvement of AIF in MPTQ-mediated cell death in neuro 2a cells we utilized both western blot analysis and immunocytochemistry. Result from western blot analysis didn’t show any visible changes in AIF level between MPTQ treated neuro 2a cells and its corresponding controls (Figure 11A). However, immunofluorescence analysis demonstrated granular pattern of AIF in the cytoplasm of untreated and treated cells but AIF was detected more in the nucleus of MPTQ treated neuro 2a cells than untreated cells (Figure 11B). Analysis of immunofluorescence image under similar settings demonstrated approximately 13% of the cells were positive for mild nuclear AIF immunostaining in controls whereas more than 80% of the cells were positive for nuclear AIF immunosignal in MPTQ treated neuro 2a cell (Figure 11C). The level of nuclear AIF immunosignal was approximately 4-fold more in MPTQ treated neuro 2a cells than untreated cells and are statistically highly significant (p<0.001) (Figure 11D). It should be noted that nuclear translocation may not alter the total cellular level of AIF. In addition, we observed an increased immunosignal of AIF in the cytoplasm of MPTQ treated cells than untreated cells. This could be due to the easy interaction of AIF to its antibody outside the mitochondria in MPTQ treated neuro 2a cells than inside the mitochondrial membrane in untreated cells. To validate the nuclear translocalization of AIF, western blot analysis was employed on cytosolic and nuclear fractions of untreated and MPTQ treated neuro 2a cells. Our results show similar AIF and GAPDH level in the cytosolic fraction of MPTQ treated and untreated cells (Figure 11E and F). In nuclear fraction, approximately 8-fold increased AIF level is observed in MPTQ treated neuro 2a cells than untreated cells (Figure 11F). However, such dramatic change of nuclear AIF level is not captured on our western blot (Figure 11E, lower panel). We found small amount of cytoplasmic contamination in our nuclear fraction and which was more in untreated cells than MPTQ treated cells (Figure 11E, lower panel). This could be partially because of unequal cell density between untreated and treated neuro 2a cell. However, after normalizing the AIF density by GAPDH and histone H3 protein, a significant increased nuclear AIF level was established (Figure 11F) suggesting the involvement of AIF as a signature for the activation of caspase-independent apoptotic pathway in MPTQ treated neuro 2a cell deaths.

## Discussion

The results from the present study clearly demonstrated that, MPTQ, a quinoline derivative compound, a structural analogue of ellipticine is cytotoxic not only to mouse neuro 2a neuroblastoma cells but also to human SH-SY5Y neuroblastoma cells in a dose and time-dependent manner. Furthermore, our results for the first time illustrated the mechanisms associated with MPTQ-mediated cytotoxicity in neuroblastoma cells. Although the reports on anticancer property of MPTQ has been very limited, but MPTQ inhibited proliferation of various leukemic cells [20], [21], melanoma cell [19] and mouse breast carcinoma cells [21]. Till today, a single report suggests the cytotoxic effect of MPTQ on neuro 2a neuroblastoma cell with approximately 40% growth inhibition after 48 hrs [19]. Our results from Live-Dead assay and MTT assay showed more than 99.8% neuro 2a and 90% SH-SY5Y neuroblastoma cell deaths upon MPTQ treatment (Figure 2 and 3 respectively). The suggestive mode of antiproliferative action was attributed to its DNA intercalating property [18], [19]. Our results also suggested that, anticancer effect of MPTQ on neuroblastoma cells continued over a long period of time. A similar result was also observed in MPTQ treated various leukemic cells [20]. We have not tested the unwanted toxic side effects of MPTQ. However, ellipticine, a structural analogue of MPTQ has limited toxic side effects and absolute no haematological toxicity [15]. Our previous study on MPTQ treated mice had no side effects from the drug during the course of treatment and specifically reduced the tumour load from a breast cancer cells xenografts [21], suggesting a better bioavailability of MPTQ to tumour mass *in vivo*. A similar result was also observed in subcutaneous graft of B16 melanoma cells [19]. Taken together, MPTQ exhibits strong anticancer property which can be harnessed positively for treating neuroblastoma patients.

Mechanisms associated with MPTQ-mediated neuroblastoma cell toxicity are not known. DNA intercalating agents are known to induce nuclear DNA breaks by altering cleavage/religation equilibrium of topoisomerase II [59]. DNA fragmentation represents severe form of cell damage and fragmentation of nuclear DNA into nucleosome-size is a key feature of apoptosis [60], [61]. Here we found that, MPTQ treatment induces DNA double strand breaks of nucleosome-sized fragments in a dose-dependent manner. Nuclear DNA fragmentation was also reported in MPTQ-treated leukemic cells [20]. Thus, our results for the first time indicated that MPTQ-mediated neuroblastoma cell death might be activating and employing cellular apoptotic machineries. Never the less, internucleosomal DNA fragments are also seen in some early necrotic cells [62]. Apoptotic cells contain blunt end, 3′and 5′ overhang DNA breaks where as necrotic cells have only 5′ overhang DNA breaks [32]. Terminal deoxynucleotidyl transferase (TdT) catalyzes the incorporation of deoxynucleotides to the free 3′-hydroxyl terminus of DNA. For DNA double stranded breaks, TdT-mediated DNA extensions are most efficient when they have 3-overhangs [33]. Since apoptotic cells have only 3′ overhangs but not in necrotic cells we validated MPTQ-mediated DNA fragmentation using TUNEL (terminal deoxynucleotidyl transferase-mediated dUTP nick end-labelling) assay. Our results clearly show significant increased TUNEL positive cells in MPTQ treated neuro 2a cells than corresponding control cells (Figure 4). At present, there is no report showing TUNEL positive cells in MPTQ-mediated cytotoxicity in any cancer cells. However, TUNEL positive cells were considered apoptotic in ellipticine treated A549 lung carcinoma cells [63]. Thus, increased nucleosomal-size nuclear DNA fragmentation and increased TUNEL positive cells in MPTQ treated neuro 2a cells strongly suggests the possible involvement of apoptosis.

Pathways related to apoptosis have never been studied in MPTQ-mediated cell death in neuroblastoma. DNA intercalating agents like ellipticine are potent genotoxic agents and induces nuclear DNA fragmentation. DNA double strand breaks are one such kind and are potent activator of ataxia-telangiectasia mutated (ATM) protein [34], [36]. DNA DSBs regulates the changes in ATM protein that eventually activates its kinase domain to phosphorylate Ser1981 in human and Ser1983 in mouse by intermolecular autophosphorylation [39]. Since MPTQ is a structural analogue of ellipticine and DNA damage was seen in MPTQ treated neuroblastoma cells, activation of ATM was hypothesized. In our current study, we found significant increased nuclear phosho-ATM (Ser1983) protein by immunocytochemistry as well as in the nuclear fraction by western blot analysis in MPTQ treated cells than untreated cells indicating its participation to genotoxic lesions induced by MPTQ. So far there is no report stating the activation of ATM in any MPTQ treated cancer cells. Thus, activation of ATM strongly support our finding on the induction of DNA double strand breaks in MPTQ treated neuroblastoma cells and might play a key role in the activation of apoptotic cascade by activating downstream target proteins.

The p53 tumour suppressor protein plays a vital role in regulating cells with damaged DNA [64]. Upon DNA damage, p53 is phosphorylated at several sites in its transactivation domain, including at Ser15 and Ser20 [65]. Activated ATM phosphorylate p53 at Ser15 directly and indirectly at Ser20 [41], [42], [43], [44]. Here, we found increased phosphorylation of p53 at Ser15 in MPTQ treated than untreated neuroblastoma cells without any detectable changes in total p53 level. We also found increased phosphorylation of p53 at Ser20 in MPTQ treated than untreated neuroblastoma cells by immunocytochemistry (data not shown). Activated p53 at ser15 was found to be confined only in nuclear compartments of MPTQ treated neuro 2a cells indicating its function only restricted to nucleus. Recently, Sharma et al., have shown that MPTQ upregulates p53 in a leukemic cell line [21]. However, posttranslational modifications of p53 were not shown in their study as we have also not examined other posttranslational modifications of p53. Kuo et al., have shown that p53 protein level was increased in ellipticine treated HepG2 cells and MCF-7 breast cancer cells [66], [67]. Furthermore, ellipticine had been shown to stabilize mutated p53 and restored the lost function of mutated p53 [68]. Thus, activation of p53 validates the function of activated ATM in MPTQ treated neuro 2a cells and further suggests the genotoxic effect of MPTQ on neuro 2a neuroblastoma cells. Like ATM, activation of p53 activates several downstream targets those are associated with mitochondrial dysfunction and initiate intrinsic apoptosis pathway. Bax (Bcl2-associated×protein) is an immediate early p53-responsive gene [69]. In response to apoptotic stimuli, Bax undergoes conformational changes, oligomerizes and inserts into the mitochondrial outer membrane (MOM) [70]. Oligomerization of Bax leads to the permeabilization of MOM and the release of cytochrome c, which in turn activates intrinsic apoptotic pathway [71]. Based on this, we studied Bax expression in MPTQ treated neuroblastoma cells to understand the function of p53 activation and possible involvement of mitochondrial apoptosis pathway in our study. MPTQ treated neuroblastoma cells demonstrated significant increased Bax expression than untreated cells. In addition, cytoplasmic Bax was found to be shifted from diffused distribution in control cells to punctuated cytoplasmic localization in MPTQ treated cells indicating possible oligomerization of Bax protein. A similar Bax distribution was also reported in staurosporine treated Cos-7 cells [72]. Overexpression of Bax can accelerate cell death in response to various apoptosis stimuli [47]. In addition, structurally related ellipticine increased the expression of Bax in MDA-MB-231 human breast cancer cells [73]. Collectively, MPTQ-mediated overexpression and punctuated cytoplasmic distribution of Bax in neuro 2a neuroblastoma cells suggest the activation of p53-dependent mitochondrial apoptosis pathway.

Two important components of mitochondrial apoptosis pathway include caspase-dependent and independent pathways [74]. In this report, we observed consistent activation of caspase-9 in the form of a ∼35 kDa protein (cleaved caspase-9) but not caspase-2 in MPTQ treated neuroblastoma cells. Both caspase-9 and caspase-2 are well established initiator caspases for intrinsic apoptotic pathway [75] suggesting for the first time that, MPTQ treated neuroblastoma cell death is mediated through only caspase-9 driven intrinsic apoptotic pathway. Activation of caspases is a hallmark feature of apoptosis. We also observed cleaved caspase-3 and caspase-7 products (activated) in MPTQ treated neuroblastoma cells. Both caspase-3 and caspase-7 are target of caspase-9 and are executor caspases of cellular intrinsic apoptotic pathway [76], [77]. In some instances, activation of p53 activates extrinsic apoptosis pathway by proteolytic activation of caspase-8 and -10 [21]. Since caspase-10 gene is absent in mice [75] we explored the proteolytic activation of caspase-8 in our study. Proteolytic products of caspase-8 were not observed in MPTQ treated neuro 2a cells. Thus, MPTQ-mediated neuroblastoma cell death involves only intrinsic but not extrinsic apoptotic pathway. Recently Sharma et al., have shown that MPTQ induced both intrinsic and extrinsic apoptotic pathway in K562 myeloid leukemia cell line suggesting different cell types may employ alternative apoptotic pathways in response to MPTQ treatment. Activated caspase-3 was reported to be present in nucleus and target nuclear protein such as PARP [78]. Our result showed significant increased presence of caspase-3 in the nucleus of MPTQ treated neuroblastoma cells than untreated cells. In addition, extensive cleaved PARP was also seen only in MPTQ treated neuroblastoma cell lysates by western blot analysis and only in the nucleus of MPTQ treated neuroblastoma cells by immunocytochemical analysis (see Figure 9). Unlike our finding, PARP proteolysis was not seen in MPTQ treated K562 myeloid leukemia cell line in spite of the activation of caspase-3 [21]. This could be because of difference in cell lines used or weaker activation of caspase-3 in MPTQ treated K562 myeloid leukemia cells. Furthermore, proteolysis of PARP by cysteine proteases such as caspases differentiated apoptosis from necrosis in MDCK cells. PARP remain intact during necrosis [62]. Thus, proteolysis of PARP along with proteolytic activation of caspases strongly suggests the involvement of apoptosis in MPTQ-mediated cell death in neuroblastoma cells.

Another factor which gets affected to mitochondrial outer membrane permeabilization is apoptosis inducing factor (AIF). Translocation of AIF from mitochondria to cytoplasm and nucleus provokes chromatinolysis and caspase-independent apoptosis [58]. In our study we found only a 67 kDa band with no alteration in amount in both untreated and MPTQ treated neuroblastoma cells indicating MPTQ has no effect on the induction of AIF expression. However, the results from immunocytochemistry and western blot analysis of cytoplasmic and nuclear fraction demonstrated significant increased AIF signal in the nucleus of MPTQ treated neuroblastoma cells. It has been reported that, AIF undergo proteolysis to form a 57 kDa truncated AIF prior to nuclear translocation [79], [80]. However we did not see 57 kDa AIF products in any isolate of our study. It is not clear whether AIF proteolysis is critical for the translocation of AIF into nucleus and its nuclear chromatinolysis activity [58], [81], [82]. In parallel to our results, only a 67 kDa AIF was observed in both cytoplasmic and nuclear extract of curcumin-induced apoptosis in human foreskin-derived fibroblasts. Inhibitors for pan-specific caspases and calpain failed to restrict AIF nuclear translocation in their study suggesting such molecular events are not always necessary for AIF function in response to apoptosis [81]. Moreover, Bax-mediated VDAC activation and induction of ceramide synthesis were involved in the release of AIF from mitochondria [81]. Since Bax is overexpressed in our study, we anticipate AIF nuclear translocation might be independent of its proteolytic activation and depend on soluble AIF present in the mitochondrial intermembrane space.

**Figure 12:**
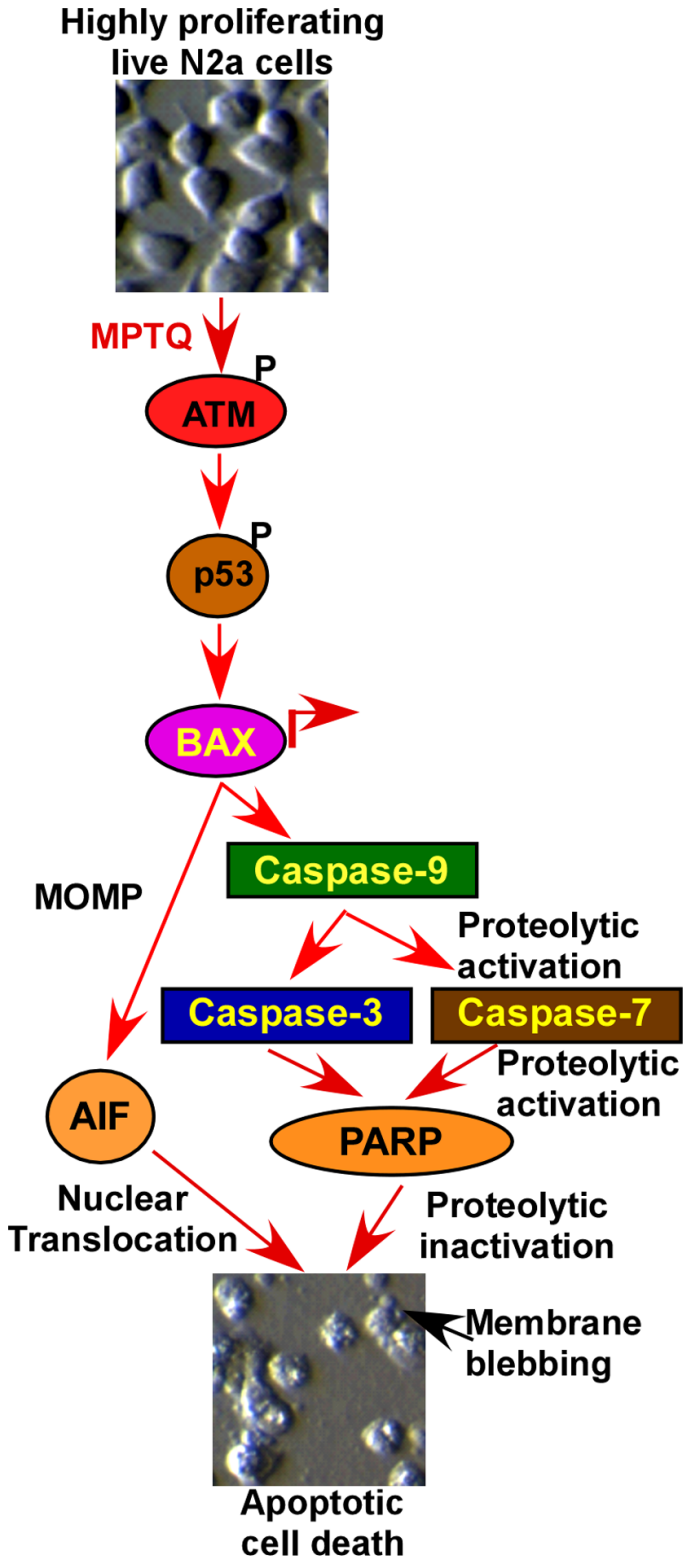
Working model of MPTQ-mediated apoptosis in neuro 2a neuroblastoma cells. MPTQ activates ATM (an indicator of DNA double strand breaks) and p53. MPTQ treatment also upregulates Bax protein level which activates caspase-dependent intrinsic apoptosis pathway by activating caspase-9 followed by caspase-3 and -7 which in turn inactivates PARP. Caspase-independent intrinsic apoptosis pathway was also activated by nuclear translocation of AIF. MOMP = mitochondrial outer membrane permeabilization.

In conclusion, the results presented in this paper demonstrated novel mechanisms associated with cytotoxic property of MPTQ on neuroblastoma cells. The mode of MPTQ-mediated cell death has been illustrated as a schema in figure 12. However, salient features of our study which report for the first time are 1) MPTQ induces neuroblastoma cell deaths not only in mouse neuro 2a cells but also in human SH-SY5Y cells. 2) MPTQ-mediated neuroblastoma cell death activates apoptotic pathway through ATM-p53-Bax-dependent mitochondrial apoptosis pathway. 3) MPTQ activates intrinsic apoptotic pathway but not extrinsic apoptosis pathway. 4) MPTQ also engaged caspase independent intrinsic apoptotic pathway by AIF nuclear translocation (Figure 12). Such multi-modal induction of apoptosis pathways by MPTQ strongly suggests its potential use as a new anticancer drug for the management of neuroblastoma.
